# Genomic and Immunoinformatics Insights Into a Bovine‐Derived *Brucella abortus* S19 Field Strain: Adaptations Impacting Vaccine Efficacy

**DOI:** 10.1002/vms3.70593

**Published:** 2025-11-03

**Authors:** Ali Arslan, Emre Aktas, Osman Ugur Sezerman, Tulin Ozbek

**Affiliations:** ^1^ Department of Molecular Biology and Genetics Graduate School of Science & Engineering Yildiz Technical University Istanbul Turkey; ^2^ Faculty of Art and Science Molecular Biology and Genetics Yıldız Technical University Istanbul Turkey; ^3^ Department of Biostatistics and Bioinformatics Institute of Health Sciences Acibadem Mehmet Ali Aydinlar University Istanbul Turkey

**Keywords:** antigenic variation, bacterial virulence factors, *Brucella abortus* S19, comparative genomics, *in silico* analysis, SNP analysis, whole‐genome sequencing

## Abstract

*Brucella abortus* S19 is a widely used live attenuated vaccine strain for bovine brucellosis control; however, its long‐term efficacy is challenged by genomic plasticity and adaptive mechanisms. This study presents a comprehensive comparative genomic and immunoinformatics analysis of a field strain (*B. abortus* S19, BAS19) isolated from an aborted cattle placenta 3 years post‐vaccination in Erzurum, Turkey. Whole‐genome sequencing was performed using Oxford Nanopore Technology, followed by genome assembly, functional annotation and comparative analyses against the reference strain (*B. abortus* S19, BAR19). Genomic variations, including 1153 single nucleotide polymorphisms (SNPs), 120 insertions and 2501 deletions, were identified. Annotation revealed 772 hypothetical proteins in BAS19 compared to 604 in BAR19, with distinct differences in virulence‐associated genes. Immunoinformatics analysis of 95 outer membrane proteins (OMPs) indicated significant antigenic variation, with 47 proteins exhibiting epitope loss and 11 displaying novel epitope gains. Beta‐barrel structure prediction demonstrated a reduction in structural stability, with nine OMPs losing beta‐barrel motifs, potentially influencing host‐pathogen interactions. These findings highlight key genomic adaptations in BAS19 that may influence its immunogenic properties and vaccine efficacy. The results contribute to a deeper understanding of *B. abortus* genomic diversity, providing insights for the rational design of improved vaccines and therapeutics tailored to regional epidemiological needs.

## Graphical abstract

This study presents a comprehensive genomic and immunoinformatics analysis of a bovine‐derived *Brucella abortus* S19 field strain, revealing significant antigenic variations and virulence adaptations impacting vaccine efficacy. These findings provide insights for designing improved vaccines tailored to regional epidemiological needs.

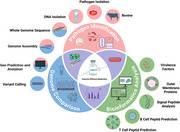

**Abbreviations**
BAR19: *Brucella abortus* S19 reference strain
BAS19: *Brucella abortus* S19 field strain
bp: base pairs
CDS: coding DNA sequence
EC: Enzyme Commission
FAST5: raw signal data format for nanopore sequencing
FASTQC: fast quality control
GC Content: guanine–cytosine content
INDEL: insertion and deletion
KEGG: Kyoto Encyclopaedia of Genes and Genomes
L50: contig count at which 50% of the genome is covered
MDS: molecular dynamics simulation
BoLA: Bovine Leukocyte Antigen
BoLA I/II: Bovine Leukocyte Antigen I/II
N50: the contig length at which 50% of the genome is assembled
OMP: outer membrane protein
ONT: Oxford Nanopore Technology
ORF: open reading frame
PGFam: PATRIC Cross‐Genus Protein Families
PLFam: PATRIC Genus‐Specific Protein Families
PRED‐TMBB2: Prediction of Transmembrane Beta‐Barrel Proteins 2
Qubit: Fluorometric DNA quantification tool
RASTtk: Rapid Annotation using Subsystem Technology toolkit
rRNA: ribosomal RNA
SNP: Single Nucleotide Polymorphism
tRNA: transfer RNA
VCF: variant call format
WGS: Whole‐Genome Sequencing

## 1 Introduction

*Brucella abortus* is a Gram‐negative, intracellular pathogen with zoonotic potential, posing a significant threat to both human and animal health due to its ability to establish chronic infections (Atluri et al. 2011; Ahmed et al. 2016; Jiao et al. 2021; Christopher et al. 2010). Infected animals experience abortion, infertility and reduced milk production, leading to substantial economic losses in the livestock sector worldwide (Corbel 2006; Godfroid et al. 2005). In humans, *Brucella spp*. infection results in brucellosis, a debilitating disease characterized by fever, myalgia and fatigue, which, if left untreated, can become chronic (Pappas et al. 2006). This highlights the pathogen's critical impact on both veterinary and public health.

The immune system defends the body against pathogens through two main mechanisms: innate immunity, which provides a rapid using physical barriers, phagocytes and natural killer cells but non‐specific response, and adaptive immunity, which is highly specific and establishes immunological memory for faster responses upon re‐exposure with T and B cells (Murphy and Weaver 2016). Vaccines stimulate adaptive immunity by presenting antigens, preparing the immune system for rapid and effective responses (Plotkin 2014). Veterinary vaccines in the field predominantly utilize live attenuated formulations, which elicit strong immune responses but require caution in immunocompromised animals (Ellis 2001). Notable examples include *B. abortus* S19, RB51 and *B. abortus* Rev‐1, which are widely used to control brucellosis in livestock. Vaccines activate cellular immunity (CD8+ cytotoxic T cells eliminate infected cells, while CD4+ helper T cells aid B cells in antibody production) and humoural immunity (B cells produce antibodies that neutralize pathogens) (Murphy and Weaver 2016).

The *B. abortus* S19 strain is a live attenuated vaccine strain widely used in brucellosis control programs due to its reduced virulence and strong immunogenic properties (Schurig et al. 2002). However, despite its widespread use, abortion rates in infected herds remain between 20% and 50%, indicating that current vaccines are not entirely effective in preventing reproductive losses (Poester et al. 2013). These losses result in an estimated annual economic burden exceeding $3.4 billion globally (Eşki et al. 2021). In Turkey, particularly in the Eastern Anatolia region, *Brucella*‐related reproductive losses and associated treatment costs contribute to an estimated annual loss of approximately $50 million, threatening rural economies dependent on livestock farming (Köroğlu et al. 2019). Furthermore, the genetic plasticity and adaptive mechanisms of *B. abortus* contribute to the progressive decline in the efficacy of the S19 vaccine over time (Moreno et al. 2022). In rare cases, the live attenuated S19 strain has been reported to colonize the host and cause abortion, further underscoring the necessity for more comprehensive genomic and immunogenic studies (Raghun et al. 2018).

This study integrates whole‐genome sequencing (WGS), comparative genomics and immunoinformatics approaches to comprehensively analyse the genomic structure, virulence factors and immunogenic properties of *B. abortus* S19 (BAS), a field strain isolated from the placenta of a cattle that experienced abortion 3 years post‐*B. abortus* S19 vaccination in Erzurum, Turkey. The comparative genomic analysis includes *B. abortus S19* (BAR) as the reference strain, obtained from the NCBI RefSeq database (NC_010742.1 ChrI and NC_010740.1 ChrII), enabling a comprehensive evaluation of genomic variations, virulence‐associated determinants and immunogenicity between the field and reference strains (Moreno et al. 2022; Liu et al. 2022; Godfroid et al. 2005). The in vitro analyses include bacterial culture conditions, genomic DNA (gDNA) isolation and sequencing using Oxford Nanopore Technology (ONT), while in silico analyses employ state‐of‐the‐art bioinformatics pipelines for genome assembly, gene annotation, variant detection, comparative genomics, virulence factor prediction and epitope mapping (Kolmogorov 2019; Brettin et al. 2015; Danecek et al. 2020; Triebel et al. 2023). Computational tools such as Minimap2, Flye, RASTtk, PRED‐TMBB2 and NetBoLApan are utilized to characterize the antigenic landscape of BAS and predict potential B and T cell epitopes for vaccine design (Li 2018; Tsirigos et al. 2016; Jespersen et al. 2017; Jurtz et al. 2017). By integrating advanced bioinformatics and immunoinformatics methodologies, this study aims to identify genomic variations that influence virulence and immunogenicity, thereby laying the groundwork for the development of improved vaccine formulations and precision therapeutics tailored to regional epidemiological needs (Weerarathna et al. 2024; Kafle and Ojha 2024). The findings from this research provide a comprehensive genomic and immunogenic profile of BAS, contributing to a better understanding of host‐pathogen interactions and facilitating the rational design of next‐generation vaccines and immunotherapeutic strategies (Fisch et al. 2021; Saha and Raghava 2006; Teufel et al. 2022; Dawood et al. 2023).

## 2 Materials and Methods

### 2.1 In vitro Analysis

#### 2.1.1 Growth Condition of *B. abortus S19* and Genomic gDNA Isolation

The BAS field strain was isolated from the placenta of a cattle that experienced abortion 3 years after *B. abortus* S19 vaccination in Erzurum. Following isolation, the strain was cultured on Blood agar plates at 37°C under a 5% CO_2_ atmosphere. After incubation, a single colony was selected and cultured overnight in 10 mL of Brain Heart infusion (BHI) broth at 37°C with 5% CO_2_ to obtain sufficient biomass for DNA isolation. The liquid culture was centrifuged at 20,000 × *g* for 5 min at 4°C to remove the supernatant, and the resulting pellet was resuspended in lysis buffer. This process facilitated the disruption of cell membranes and walls, allowing the release of gDNA. The gDNA extraction was performed following the protocols of the ZymoBIOMICS DNA MiniPrep Kit. The purity of the isolated gDNA was assessed using a Nanodrop 2000 spectrophotometer, ensuring a 260/280 ratio of ≥1.8. DNA concentration was precisely measured with the Qubit High Sensitivity 2.0 dsDNA Assay. The high‐quality gDNA obtained served as the foundation for WGS and subsequent bioinformatics analyses (Akmayan et al. 2024).

#### 2.1.2 DNA Purification and Whole Genome Sequencing

The ONT SQK‐LSK109 Sequencing Kit was utilized for WGS. The extracted gDNA underwent 3′ and 5′ end repair using the NEBNext Ultra II End Prep enzyme mix and NEBNext FFPE DNA Repair Mix, followed by purification with AMPure XP beads. Sequencing adapters were ligated to the DNA using Quick T4 DNA Ligase. Library preparation was completed in accordance with the ONT SQK‐LSK109 protocol The sequencing process was conducted on a MinION device using Flowcell v9.4.1, and the resulting data were stored in FAST5 format for genome assembly and annotation analyses. To enhance sequencing efficiency, DNA concentration was optimized based on a 10 ng/µL cut‐off value, as determined by the Qubit High Sensitivity Assay. The obtained data were subsequently employed for genome annotation and variant analysis (Erturk et al. 2023).

### 2.2 In silico Analysis

#### 2.2.1 Genome Assembly

The genomic data of BAS obtained after sequencing were subjected to basecalling using the MinKNOW interface and subsequently converted into FASTQ format (S. Wang et al. 2020). Basecalling is a critical step in transforming raw sequencing reads into biologically meaningful nucleotide sequences (Napieralski and Nowak 2022). FASTQC was employed to assess sequence quality and filter out low‐quality reads (Wingett and Andrews 2018). A stringent quality threshold of Q‐score ≥10 was applied to ensure high‐confidence data selection (Jauhal and Newcomb 2021; Payne et al. 2018). Long‐read sequences were subsequently processed for error correction and optimal genome assembly using Flye v2.8.3 (Kolmogorov 2019), an assembler specifically optimized for long‐read sequencing technologies, ensuring high accuracy in complex repeat regions. Assembly quality was assessed using the N50 metric (≥ 50,000 bp), and a minimum sequencing depth of 50× was maintained to eliminate low‐coverage areas. The final high‐quality genome assembly was prepared for downstream functional genomic analyses.

#### 2.2.2 Gene Prediction and Annotation

To annotate the structural and functional components of the BAS genome, RASTtk (Rapid Annotations using Subsystems Technology) was utilized (Brettin et al. 2015). RASTtk facilitates bacterial genome annotation by leveraging an extensive reference database to predict gene function. Coding sequences (CDS) were identified using Glimmer (Delcher et al. 2007), which employs a Markov model‐based algorithm to accurately detect open reading frames (ORFs) ≥ 100 bp, minimizing false positives. Transfer RNA (tRNA)‐encoding genes were detected using tRNAscan‐SE (Chan et al. 2019). The annotation results were submitted to GenBank (SRR14268008) for further genomic analysis.

#### 2.2.3 Variant Calling

To identify genomic variations, the BAS genome was aligned against the reference BAR genome (NC_010742.1 ChrI and NC_010740.1 ChrII) using Minimap2 (Li 2018), which is optimized for long‐read sequence alignment and ensures high‐accuracy reference–genome comparisons. The alignment data were processed using SAMtools (Danecek et al. 2020) and bcftools, and genetic variants, including single nucleotide polymorphisms (SNPs), insertions and deletions (INDELs) and frameshift mutations, were identified and stored in VCF format. Functional annotation of the detected variants was performed in RStudio (Knaus and Grünwald 2017) to elucidate the biological significance of the mutations, particularly their impact on virulence factors (Supporting Information S1).

#### 2.2.4 Comparative Genomics between BAS and BAR Genomes

Comparative genomic analysis was conducted to investigate evolutionary relationships and genetic variations between BAS and BAR genomes. To ensure the reliability of the analysis, reference genome sequences were retrieved from NCBI RefSeq (Pruitt et al. 2004). Genomic variations were identified using GenVar, which allowed for the detailed characterization of SNPs, INDELs and frameshift mutations. In addition, ORF prediction was performed using ORF Finder (Rombel et al. 2002) to examine the impact of these variations on gene function.

#### 2.2.5 Determination of General Virulence Factors

To identify bacterial virulence‐associated factors, the VFDB (Virulence Factor Database) available on the PATRIC platform was queried (Mao et al. 2014; Liu et al. 2022). Comparative analysis between BAS and BAR genomes facilitated the identification of defective bacterial virulence factors, which play a crucial role in host‐pathogen interactions (Liu et al. 2022). This analysis provided insights into the potential alterations in pathogenesis mechanisms between the two strains.

#### 2.2.6 Prediction of Outer Membrane Proteins and Beta‐Barrel Structures

Outer membrane proteins (OMPs) enhance immune responses, particularly in *B. abortus* vaccines (Pasquevich et al. 2019). After the annotation of the previously sequenced BAS19 sample, the obtained GenBank data were analysed by comparing them with the proteins present in the reference database. Reference proteins were obtained from the UniProt database (entry number 430066). The BAS genome was screened for 95 OMPs, and a comparative analysis was performed on these proteins (Supporting Information S4).

#### 2.2.7 Bacterial Antigen Identification and Signal Peptide Analysis

A subset of 30 bacterial virulence‐associated proteins was selected as potential antigen candidates, and multiple sequence alignments were performed using Clustal Omega (Sievers and Higgins 2014) (Supporting Information S2). To assess their secretory potential, SignalP 6.0 was employed to predict the presence of signal peptides (Teufel et al. 2022). Proteins containing signal peptides were further evaluated for their involvement in BoLA Class I and II antigen presentation pathways, aiding in the identification of immunogenic targets (Pishesha et al. 2022). This approach facilitated a comprehensive immunological comparison between BAS and BAR strains.

#### 2.2.8 Prediction of B and T Cell Epitopes for Bacterial OMPs

The proteins identified in previous stages were compared with reference data, and BoLA I, BoLA II and B‐cell predictions were analysed to assess the potential impact of relevant INDEL or SNP variations on the immune response. In cattle, the major histocompatibility complex (MHC) is referred to as the bovine leukocyte antigen (BoLA); thus, BoLA class I and II correspond to MHC class I and II molecules.To identify immunodominant epitopes, BepiPred‐2.0 and ABCpred were utilized to predict B cell epitopes within the 30 bacterial virulence‐associated proteins. BepiPred‐2.0 employed a threshold of 0.5 for epitope prediction, while ABCpred, based on artificial neural networks, analysed 16‐amino acid sequence windows to identify potential antigenic determinants (Jespersen et al. 2017; Saha and Raghava 2006). T cell epitope prediction was conducted using NetBoLApan‐I and NetBoLApan‐II, tailored for bovine BoLA antigen presentation (Jurtz et al. 2017; Macdonald et al. 2010). The predicted epitopes were mapped onto BoLA I and BoLAII molecules, facilitating their classification as potential targets for adaptive immune responses (Wu et al. 2023; Pal et al. 2022). Comparative immunological profiling between BAS and BAR strains was performed to assess epitope loss or gain, which could influence host immune recognition and pathogenesis. Given the relevance of BoLA‐DRB3 alleles in bovine immunity, epitope variations were particularly scrutinized for their implications in immune evasion and vaccine design (Fisch et al. 2021).

## 3 Results

### 3.1 In vitro Studies

#### 3.1.1 Growth Condition of BAS and Genomic gDNA Isolation

The BAS strain was cultured on Blood agar (Supporting Information S3) and subsequently propagated in liquid culture, followed by gDNA isolation in accordance with the kit protocol. Post‐isolation, the DNA concentration was measured using a Qubit device and determined to be 72.4 ng/µL.

#### 3.1.2 DNA Purification and Whole Genome Sequencing

In the initial phase of the genome assembly process, the BAS19 genome, obtained in vitro, was stored in FAST5 format. This storage approach preserved raw signal data, allowing for more flexible and detailed analyses during data processing. The BAS19 genome sequences were annotated using the RASTtk and subjected to basecalling to serve as the foundation for genome analysis. This process facilitated the conversion of raw signal data from sequencing instruments into nucleotide sequences. Subsequently, sequence quality was assessed using FASTQC, and the N50 value was determined to enhance assembly quality. Following these analyses, genome integrity was reinforced, and read depth analysis was performed to determine the frequency of each nucleotide within the reconstructed sequence. The overall read quality was measured as Q17 (Phred Score), with read depths of 57× for Chromosome I and 60× for Chromosome II. A Q‐score of 17 corresponds to a base call accuracy of approximately 98%, which is considered moderate for ONT. However, it is generally sufficient for downstream applications such as genome assembly and variant analysis. To improve data reliability, low‐quality reads were filtered and polishing steps were applied during the assembly process. Long‐read data were then assembled with high accuracy using Flye v2.8.3, yielding a reliable and comprehensive genome assembly (Grant et al. 2023). As a result, the BAS19 genome was analysed and utilized in subsequent annotation studies.

### 3.2 In silico Studies

#### 3.2.1 Genome Assembly

**FIGURE 1:**
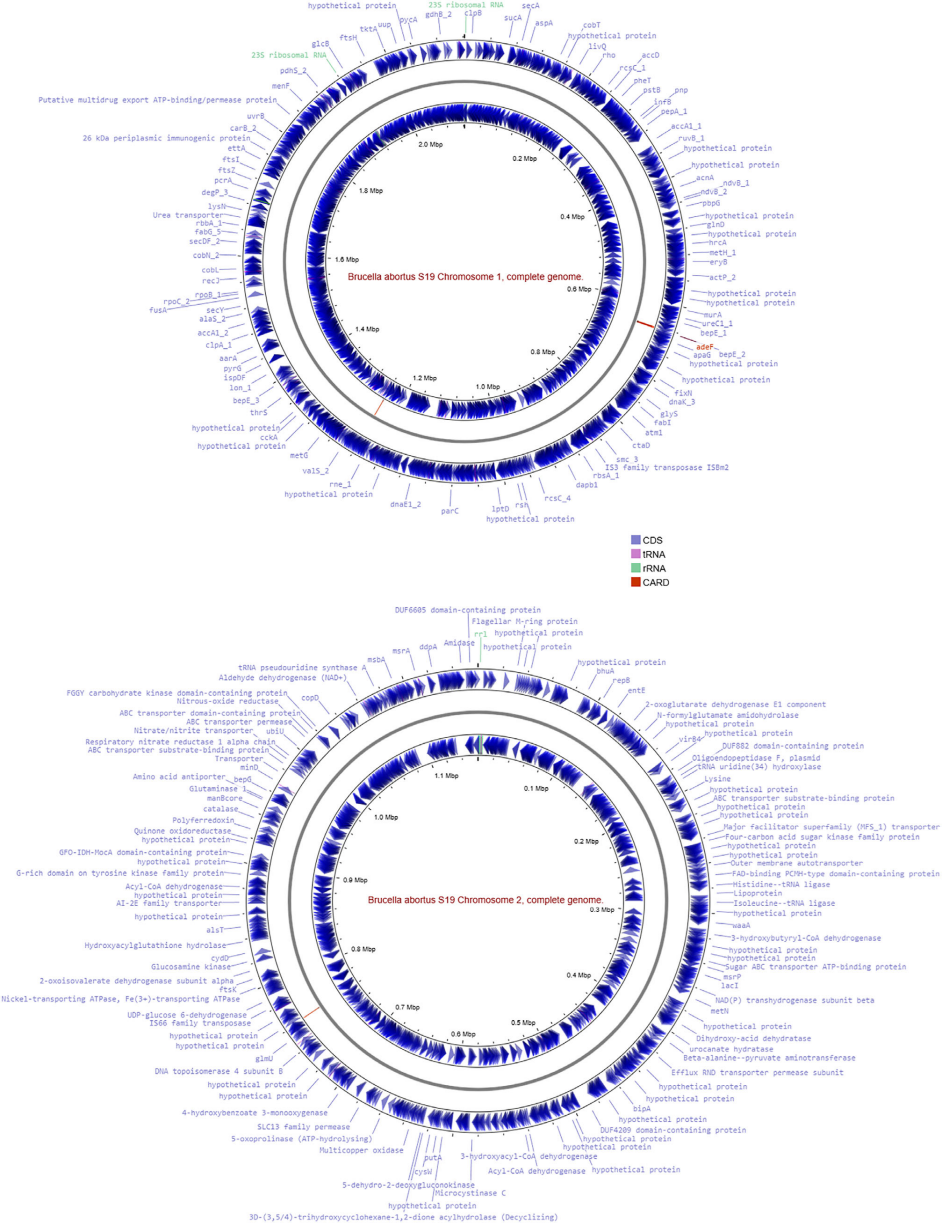
Genomic organization of BAS19 consisting of two chromosomes. Each circle represents one of the chromosomes, with inner rings showing structural genomic features such as GC content and GC skew. Outer rings display coding DNA sequences (CDS), tRNA and rRNA genes, as well as putative antibiotic resistance genes annotated using the CARD database. Coding sequences (CDS) are shown in yellow, tRNAs in red, rRNAs in green and antibiotic resistance genes (according to the CARD database) in blue. Gene annotations across the genome include hypothetical proteins, transporters, ribosomal components and potential virulence‐associated factors, providing insights into the strain's functional capacity and host adaptation.

In this study, the fundamental genomic differences between the field strain BAS19 and the reference strain BAR19 were compared. Both strains possess two chromosomes, with a GC content of 57.22%. No differences were observed in terms of plasmid content. Regarding genome length, BAS19 was determined to have a genome size of 3,282,271 bp, whereas BAR19's genome was calculated as 3,278,307 bp (Figure 1). The contig L50 value was found to be 1 for both strains, indicating a high level of genetic integrity. However, the contig N50 value was identified as 2,121,058 bp for BAS19 and 2,121,359 bp for BAR19. The number of ORFs was determined to be 4482 in BAS19 and 3291 in BAR19. In addition, BAS19 contained 89 repetitive regions, while BAR19 had 90. The number of hypothetical proteins was 772 in BAS19 and 604 in BAR19. Minor differences were observed in tRNA and rRNA gene counts. These findings highlight the genetic variations between B. abortus field and reference strains, particularly in terms of hypothetical proteins, ORF counts and repetitive regions, revealing significant genomic divergence (Table 1).

**TABLE 1:** Genomic features of BAS19 and BAR19.

Features	BAS19	BAR19
**Chromosome**	2	2
**GC Content**	57.22	57.22
**Plasmids**	0	0
**Contig L50** ^a^	1	1
**Contig N50** ^b^ **(bp)**	2,121,058	2,121,359
**Genome Length (bp)**	3,282,271	3,278,307
**Average gene length**	432.4	432.4
**ORFs**	4482	3291
**Repeat Regions**	89	90
**rRNA**	9	9
**tRNA**	53	55
**Hypothetical protein**	772	604

^a^ Contig L50; the smallest number of contigs whose sum of length is half the size of the genome.

^b^ Contig N50; Sequence information where 50% of all sequences are long.

This table compares the genomic data of the BAS19 and BAR19 strains. Chromosome represents the number of chromosomes; GC Content indicates the percentage of guanine–cytosine content; Plasmids denotes the number of plasmids; Contig L50 refers to the smallest number of contigs covering half of the genome; Contig N50 represents the base pair length at which 50% of all sequences are longer. Genome Length indicates the total genome length; Average gene length refers to the average gene size; ORFs represents the number of open reading frames; Repeat Regions denotes the number of repetitive regions; rRNA and tRNA indicate the respective RNA gene counts; and Hypothetical protein refers to proteins with unknown functions.

#### 3.2.2 Gene Prediction and Annotation

**FIGURE 2:**
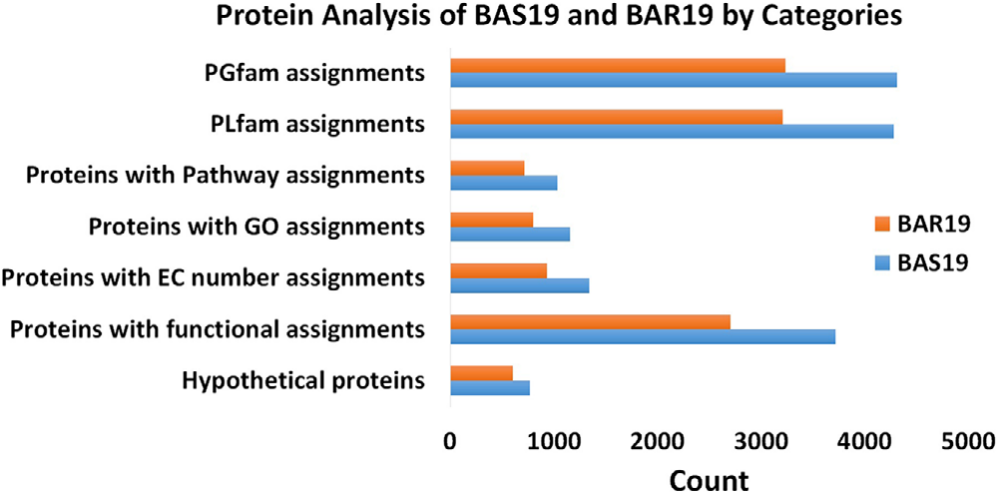
BAS19 and BAR19 protein analyses. The blue bars represent the number of proteins in the BAS19 strain, while the orange bars correspond to the BAR19 strain. The figure illustrates protein counts associated with the PATRIC cross‐species protein family (PGFam) and genus‐specific protein family (PLFam), as well as categories including metabolic pathways, Gene Ontology (GO) terms and Enzyme Commission (EC) number assignments.

The detailed annotations of the BAS19 and BAR19 genomes (Table 1, Figure 1) have provided critical insights into the genetic composition of both strains. The BAS19 genome consists of 772 hypothetical proteins and 3717 functionally assigned proteins. Among the functionally assigned proteins, 1344 proteins have been classified with Enzyme Commission (EC) numbers, 1156 proteins are associated with Gene Ontology (GO) terms, and 1037 proteins have been mapped to KEGG pathways. According to the PATRIC annotation, 4279 proteins in the BAS19 genome belong to genus‐specific protein families (PLFams), while 4313 proteins are associated with cross‐genus protein families (PGFams). In contrast, the annotation of the BAR19 genome comprises 604 hypothetical proteins and 2703 functionally assigned proteins. Among the functionally assigned proteins, 934 proteins have been categorized with EC numbers, 800 proteins are associated with GO terms, and 717 proteins have been mapped to KEGG pathways. In the PATRIC annotation, 3207 proteins in the BAR19 genome belong to genus‐specific protein families (PLFams), while 3237 proteins are assigned to cross‐genus protein families (PGFams) (Figure 2).

#### 3.2.3 Variant Calling

**FIGURE 3:**
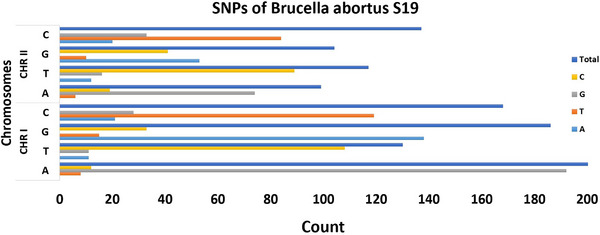
BAS19 SNP distribution across chromosomes I and II: The *x*‐axis indicates the count of SNPs, while the *y*‐axis categorizes the SNPs based on nucleotide substitutions (A, T, G, C).

Multiple SNPs were identified in the BAS genome when compared to the reference strain. A total of 1153 SNPs were detected, with 696 located on Chromosome 1 and 457 on Chromosome 2 (Figure 3). The specific nucleotide and protein‐level regions affected by these SNPs, which are irregularly distributed across both chromosomes, are documented in Supporting Information S1. Although genome alignment results indicate a high degree of similarity between the BAS and BAR genomes, distinct genetic differences were observed. As detailed in the Supporting Information, a comprehensive genomic analysis revealed variations in insertions and deletions, with an unequal distribution between the two chromosomes. In the BAS genome, all nucleotide deletions and insertions were identified as 2501 and 120, respectively, compared to BAR. These variations were distributed as 1595 deletions on chromosome 1 and 906 on chromosome 2, while insertions were recorded as 80 on chromosome 1 and 40 on chromosome 2 (Figure 3).

#### 3.2.4 Comparative Genomics Between BAS and BAR Genomes

**FIGURE 4:**
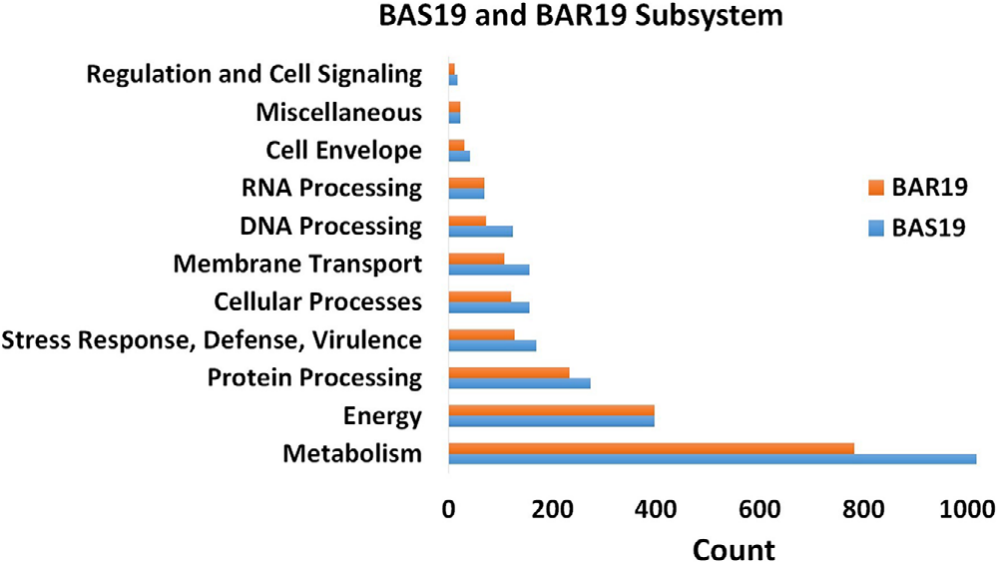
Subsystem categorization of BAS19 and BAR19 strains by using PATRIC server. The bar chart displays the subsystem classifications of metabolic and functional processes in BAS19 and BAR19 strains. Different biological functions, including metabolism, protein processing, energy, cellular processes, DNA/RNA processing and stress response, are categorized. The *x*‐axis represents the number of genes associated with each subsystem, and the *y*‐axis differentiates between the two strains.

The BAS19 genome presents a subsystem profile encompassing a total of 2485 genes, representing various biological processes and structural complexes. Key subsystems include metabolism (1018 genes), energy production (397 genes), protein processing (274 genes), stress response, defence and virulence (169 genes), cellular processes (156 genes), membrane transport (156 genes), DNA processing (124 genes), RNA processing (104 genes), cell envelope (42 genes), miscellaneous functions (28 genes) and regulation and cell signalling (17 genes) (Figure 4). Notably, the BAS19 genome contains 169 genes associated with stress response, defence and virulence, whereas the BAR19 genome exhibits a lower diversity in this subsystem, with 128 genes. BAS19 genome sequences share over 99.4% similarity with BAR19. However, the BAS19 genome is 3964 bp longer and contains 1191 more ORFs compared to BAR19.

#### 3.2.5 Determination of General Virulence Factors

**FIGURE 5:**
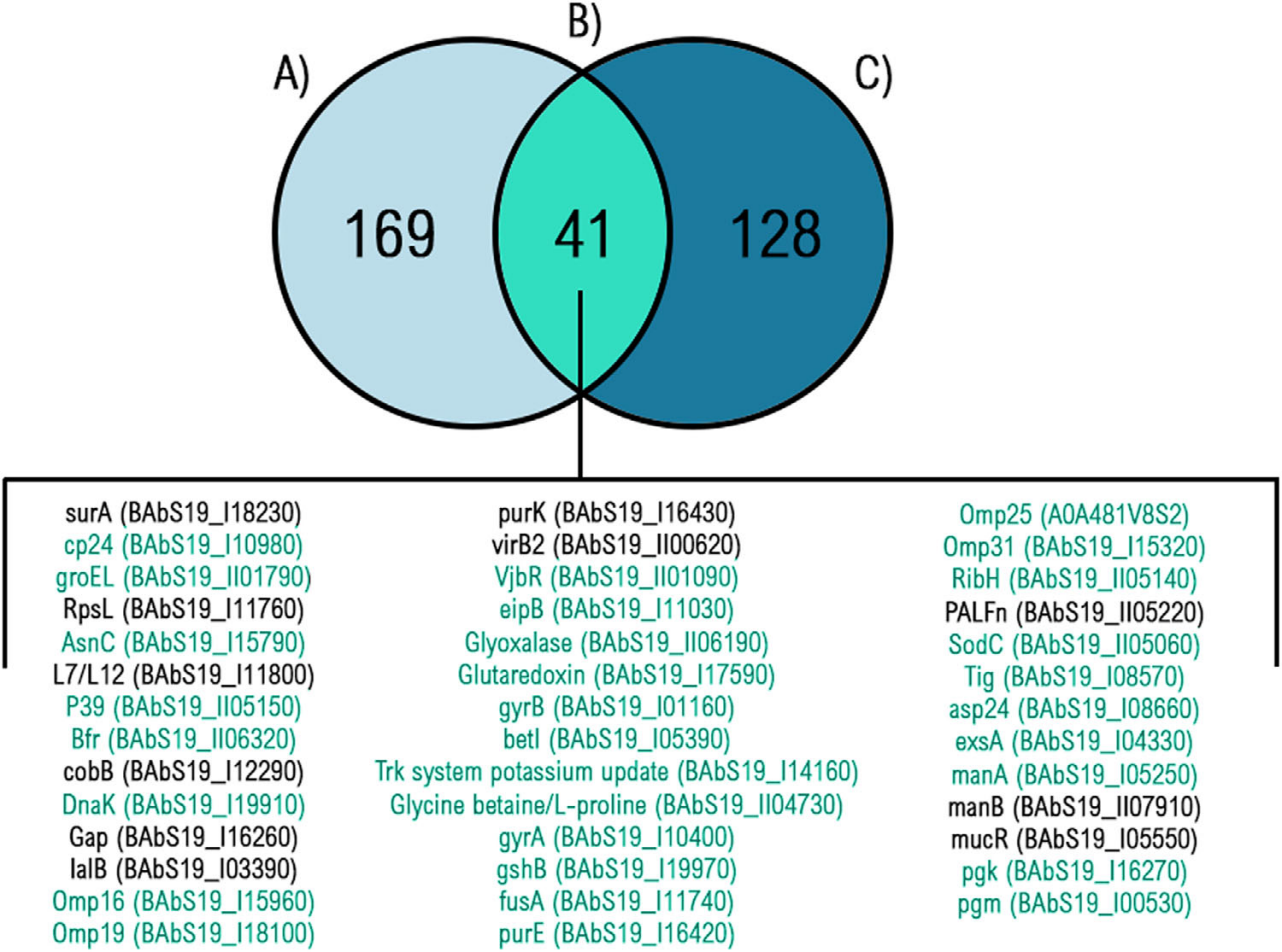
A total of 41 virulence factors were identified as differing between the BAR19 and BAS19 genomes. Among these 41 proteins, only 30 showed variations in the pairwise alignment results, and the proteins with observed variations are highlighted in green; therefore, the study continued with these 30 proteins based on the observed differences. The Venn diagram illustrates the number of unique and shared genes between BAS19 (A) and BAR19 (C) strains, with the overlapping section (B) representing the genes common to both strains.

The BAS19 genome harbours a higher number of virulence factors compared to the BAR19 genome. Specifically, while the BAR19 genome contains 128 virulence factors, the BAS19 genome exhibits 169 virulence factors (Figure 5).

Virulence factors were examined in detail, and those with immunogenic significance were identified. Virmugen refers to microorganisms with attenuated virulence, which form the basis of live attenuated vaccines. These microorganisms activate the immune system, eliciting a protective response without causing disease. Protective antigens are specific proteins that generate an immune response, particularly through antibody production or cytokine signalling, thereby providing resistance against the pathogen. Based on these proteins, we identified alternative candidate proteins that are known to be utilized in immunological studies and selected novel candidates for further investigation.

#### 3.2.6 Prediction of OMPs and Beta‐Barrel Structures

The analysis of 95 OMPs in the BAS strain has revealed significant findings regarding the presence and loss of beta‐barrel structures (Supporting Information S4).

According to the results, while BAR retains the beta‐barrel structure in 34.7% of all OMPs, this ratio decreases to 24.2% in the BAS strain. A total of nine proteins (BAbS19_I00090, BAbS19_I00100, BAbS19_I09030, BAbS19_I10940, BAbS19_II02220, BAbS19_II03380, BAbS19_II06210, BAbS19_II09770 and BAbS19_II10320) were identified as having lost their beta‐barrel structures (Table 2).

**TABLE 2:** Presents the structural assessment of OMPs based on their predicted beta‐barrel architecture and transmembrane regions.

No.	BAR			BAS				BAR			BAS		
	β‐score	#TM	Reliability	β‐score	#TM	Reliability	No.	β‐score	#TM	Reliability	β‐score	#TM	Reliability
1	**Y|1.000**	**4**	**0.989**	**N|0.000**	**2**	**0.966**	49	Y|1.000	10	0.971	Y|1.000	6	0.826
2	**Y|1.000**	**4**	**0.981**	**N|0.000**	**2**	**0.980**	50	N|0.000	—	1.000	N|0.000	—	1.000
3	Y|1.000	14	0.961	Y|1.000	4	0.943	51	N|0.000	—	1.000	N|0.000	—	1.000
4	Y|1.000	6	0.962	Y|1.000	6	0.985	52	Y|1.000	22	0.865	Y|1.000	18	0.838
5	N|0.000	—	1.000	N|0.000	—	1.000	53	N|0.000	—	0.999	N|0.000	—	0.999
6	Y|1.000	8	0.960	Y|1.000	8	0.960	54	N|0.000	4	0.962	N|0.000	2	0.980
7	Y|1.000	8	0.967	Y|1.000	6	0.902	55	N|0.000	—	1.000	N|0.000	—	1.000
8	N|0.000	—	1.000	N|0.000	—	1.000	56	N|0.000	—	1.000	N|0.000	2	0.963
9	N|0.000	—	1.000	N|0.000	—	1.000	57	N|0.000	—	1.000	N|0.000	—	1.000
10	N|0.000	—	1.000	N|0.000	—	1.000	58	N|0.000	—	1.000	N|0.000	—	1.000
11	N|0.000	—	1.000	N|0.000	—	0.993	59	Y|1.000	18	0.913	N|0.000	2	0.950
12	N|0.000	—	0.974	N|0.000	2	0.935	60	Y|1.000	6	0.817	Y|1.000	8	0.906
13	Y|1.000	26	0.859	Y|1.000	26	0.859	61	N|0.000	—	0.999	N|0.000	—	0.999
14	Y|1.000	12	0.865	Y|1.000	8	0.947	62	**Y|1.000**	**22**	**0.911**	**N|0.000**	**—**	**1.000**
15	N|0.000	—	1.000	N|0.000	—	1.000	63	N|0.000	—	1.000	N|0.000	—	1.000
16	N|0.000	—	1.000	N|0.000	—	1.000	64	Y|1.000	8	0.969	N|0.000	2	0.970
17	N|0.000	—	1.000	N|0.000	—	1.000	65	**Y|0.991**	**2**	**0.934**	**N|0.000**	**2**	**0.917**
18	N|0.000	—	1.000	N|0.000	—	1.000	66	N|0.000	—	0.952	N|0.000	—	0.951
19	N|0.000	—	0.943	N|0.000	—	0.943	67	N|0.000	—	1.000	N|0.000	2	0.951
20	N|0.000	—	1.000	N|0.000	—	1.000	68	N|0.000	—	0.999	N|0.000	—	0.999
21	N|0.000	—	0.955	N|0.000	—	0.900	69	N|0.000	—	1.000	N|0.000	—	1.000
22	Y|1.000	4	0.902	Y|1.000	4	0.897	70	**Y|0.988**	**6**	**0.964**	**N|0.000**	**2**	**0.970**
23	**Y|1.000**	**14**	**0.868**	**N|0.000**	**—**	**0.821**	71	N|0.000	—	0.998	N|0.000	—	0.997
24	N|0.000	2	0.876	N|0.000	2	0.876	72	N|0.000	—	1.000	N|0.000	—	1.000
25	N|0.000	—	0.999	N|0.000	—	0.999	73	N|0.000	—	0.988	N|0.000	4	0.787
26	**Y|1.000**	**14**	**0.945**	**N|0.000**	**—**	**0.999**	74	N|0.000	—	1.000	N|0.000	—	1.000
27	N|0.000	—	1.000	N|0.000	2	0.983	75	N|0.000	—	1.000	N|0.000	—	1.000
28	N|0.000	4	0.922	N|0.000	—	0.929	76	N|0.000	—	0.953	N|0.000	—	1.000
29	Y|1.000	8	0.861	Y|1.000	8	0.861	77	N|0.000	—	1.000	N|0.000	—	1.000
30	Y|1.000	20	0.923	Y|0.971	6	0.860	78	N|0.000	—	1.000	N|0.000	—	1.000
31	N|0.000	2	0.985	N|0.000	2	0.985	79	**Y|0.909**	**4**	**0.952**	**N|0.000**	**—**	**1.000**
32	N|0.000	—	1.000	N|0.000	—	1.000	80	N|0.000	—	0.995	N|0.000	—	0.991
33	Y|0.999	12	0.898	Y|0.999	12	0.904	81	**Y|1.000**	**50**	**0.889**	**N|0.000**	**4**	**0.900**
34	Y|1.000	8	0.949	Y|1.000	8	0.949	82	N|0.000	—	1.000	N|0.000	—	1.000
35	Y|1.000	8	0.914	Y|1.000	8	0.916	83	Y|1.000	22	0.910	Y|1.000	22	0.910
36	Y|1.000	8	0.978	Y|1.000	8	0.978	84	Y|1.000	8	0.940	Y|1.000	8	0.947
37	N|0.000	—	0.987	N|0.000	—	0.997	85	N|0.000	—	1.000	N|0.000	—	1.000
38	N|0.000	2	0.935	N|0.000	—	1.000	86	N|0.000	—	1.000	N|0.000	—	0.991
39	N|0.000	—	0.997	N|0.000	—	0.997	87	N|0.000	—	1.000	N|0.000	—	1.000
40	N|0.000	—	1.000	N|0.000	—	1.000	88	N|0.000	—	1.000	N|0.000	—	1.000
41	N|0.000	—	1.000	N|0.000	—	1.000	89	N|0.000	—	1.000	N|0.000	—	1.000
42	N|0.000	2	0.935	N|0.000	2	0.935	90	N|0.000	—	1.000	N|0.000	—	1.000
43	N|0.000	—	1.000	N|0.000	—	1.000	91	N|0.000	—	1.000	N|0.000	—	1.000
44	N|0.000	—	0.991	N|0.000	—	0.991	92	Y|1.000	12	0.936	Y|1.000	12	0.936
45	Y|1.000	4	0.950	Y|1.000	4	0.952	93	N|0.000	—	0.999	N|0.000	—	1.000
46	N|0.000	—	1.000	N|0.000	—	1.000	94	N|0.000	—	0.999	N|0.000	—	0.999
47	N|0.000	—	1.000	N|0.000	—	1.000	95	Y|1.000	14	0.905	Y|1.000	12	0.936
48	Y|1.000	8	0.861	Y|1.000	6	0.872							

The β‐score denotes the probability of a protein adopting a beta‐barrel conformation, accompanied by a confidence score reflecting the reliability of the prediction. The **#TM** column represents the predicted number of transmembrane regions, providing insights into the protein's potential membrane‐spanning characteristics. The Reliability column indicates the confidence level of these predictions, ranging from 0 (*low confidence*) to 1 (*high confidence*). Proteins classified as “**Y**” (Yes) in the β‐score column are predicted to contain a beta‐barrel structure, whereas those labelled as “**N**” (No) lack this feature.

Bold values indicate high‐confidence predictions (Reliability ≥ 0.95).

#### 3.2.7 Bacterial Antigen Identification and Signal Peptide Analysis

The comparative analysis of BAR and BAS reveals notable changes, particularly in certain critical virulence factors (Table 3). Sequence length variations were observed in a total of 30 genes, with some genes exhibiting significant length increases while others showed reductions. Among the genes with length increases, *cp24* displayed a 17.74% increase, *Omp25* increased by 2.04% and *Omp31* by 3.51%. In addition, *asp24* exhibited a 36.05% increase, *Glyoxalase* increased by 75.95% and *Glutaredoxin* by 4.55%. Moreover, *Trk system potassium uptake* showed a 4.28% increase, while *Glycine betaine/L‐proline* exhibited a 1.03% increase. Conversely, several genes displayed length reductions. *groEL* showed a 70.7% decrease, *AsnC* decreased by 2.56%, and *P39* by 82.14%. *Bfr* exhibited a 78.88% reduction, *DnaK* decreased by 92.63% and *Omp16* by 29.35%. Furthermore, *RibH* showed a 78.26% decrease, *SodC* by 53.99%, *Tig* by 2.1% and *exsA* by 35.3%. Additional length reductions were observed in *manA* (9.66%), *pgk* (54.14%), *pgm* (40.91%) and *purE* (27.9%). Some genes did not exhibit any sequence length changes. Notably, no differences were detected in the lengths of *Omp19* and *VjbR* (Table 3).

**TABLE 3:** This analysis compares specific genes and their corresponding loci in the reference genome and the observed organism.

Gene and locus	BAR Length	BAS Length	Length change (%)	Direction
***cp24* (BAbS19_I10980)**	186	219	17.74	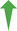
***groEL* (BAbS19_II01790)**	546	160	70.7	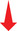
***AsnC* (BAbS19_I15790)**	156	152	2.56	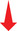
***P39* (BAbS19_II05150)**	420	75	82.14	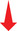
***Bfr* (BAbS19_II06320)**	161	34	78.88	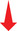
***DnaK* (BAbS19_I19910)**	624	46	92.63	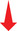
***Omp16* (BAbS19_I15960)**	276	195	29.35	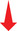
***Omp19* (BAbS19_I18100)**	249	249	0	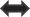
***Omp25* (A0A481V8S2)**	294	300	2.04	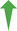
***Omp31* (BAbS19_I15320)**	342	354	3.51	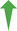
***RibH* (BAbS19_II05140)**	230	50	78.26	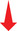
***SodC* (BAbS19_II05060)**	317	146	53.99	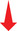
***Tig* (BAbS19_I08570)**	571	559	2.1	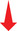
***asp24* (BAbS19_I08660)**	233	318	36.05	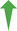
***exsA* (BAbS19_I04330)**	527	341	35.3	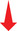
***manA* (BAbS19_I05250)**	621	561	9.66	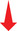
***pgk* (BAbS19_I16270)**	785	355	54.14	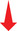
***pgm* (BAbS19_I00530)**	731	432	40.91	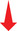
***purE* (BAbS19_I16420)**	362	261	27.9	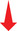
***VjbR* (BAbS19_II01090**)	336	336	0	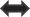
***eipB* (BAbS19_I11030)**	483	492	1.86	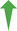
***Glyoxalase* (BAbS19_II06190)**	79	139	75.95	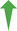
***Glutaredoxin* (BAbS19_I17590)**	88	92	4.55	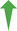
***gyrB* (BAbS19_I01160)**	813	789	2.95	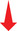
***betI* (BAbS19_I05390)**	172	170	1.16	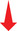
***Trk system potassium update* (BAbS19_I14160)**	467	487	4.28	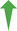
***Glycine betaine/L‐proline* (BAbS19_II04730)**	292	289	1.03	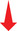
***gyrA* (BAbS19_I10400)**	871	861	1.15	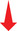
***gshB* (BAbS19_I19970)**	594	590	0.67	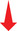
***fusA* (BAbS19_I11740)**	661	271	59	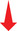

The Gene and Locus column includes the name of the examined gene and its associated locus information. Ref Length represents the gene length in the reference genome, while Org Length denotes the observed length in the studied organism. Length Change (%) indicates the percentage change relative to the reference length. In the Directions column, increases, decreases and no change are marked in bold. Change Direction specifies the direction of length variation: ‘↑’ indicates an increase, ‘↓’ represents a decrease, and ‘↔’ denotes no change.

#### 3.2.8 Prediction of B and T Cell Epitopes for Bacterial OMPs

Analyses have revealed epitope alterations across all 95 OMPs. According to BepiPred results, epitope changes were observed in 61.05% of the total proteins. Among these, epitope loss was identified in 47 proteins (49.47%), while 11 proteins (11.58%) exhibited newly acquired epitopes. The remaining 37 proteins (38.95%) showed no epitope changes, suggesting that these structures may be evolutionarily more stable. ABCpred analyses revealed a different trend, detecting epitope variations in all proteins, with no proteins remaining unchanged. In total, 48 proteins (50.53%) exhibited epitope loss, whereas 47 proteins (49.47%) displayed newly acquired epitopes (Table 4).

**TABLE 4:** This analysis presents epitope variation predictions generated using BepiPred and ABCpred methods.

OMP no.	BAR_E_Count	BAS_E_Count	Change (%)	Directions	BAR_E_Count	BAS_E_Count	Change (%)	Directions
1	78	19	76	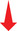	38	6	84.21	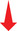
2	45	22	51	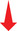	38	11	71.05	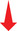
3	71	52	27	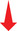	40	29	27.5	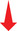
4	179	313	75	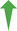	48	76	58.33	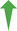
5	33	13	61	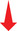	12	5	58.33	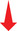
6	33	33	0	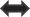	14	14	0	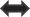
7	26	14	46	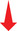	14	7	50	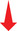
8	99	103	4	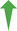	32	33	3.12	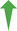
9	35	38	9	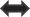	13	13	0	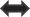
10	51	58	14	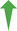	15	17	13.33	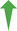
11	44	35	20	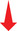	9	6	33.33	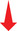
12	68	107	57	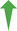	19	31	63.16	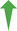
13	108	108	0	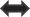	49	49	0	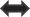
14	43	25	42	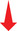	19	13	31.58	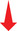
15	39	41	5	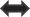	10	10	0	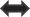
16	86	86	0	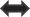	27	27	0	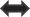
17	43	25	42	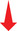	14	7	50	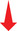
18	21	22	5	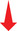	16	11	31.25	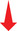
19	91	91	0	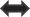	27	27	0	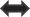
20	66	46	30	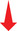	29	12	58.62	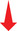
21	72	63	12	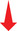	26	20	23.08	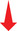
22	100	100	0	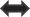	28	28	0	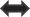
23	64	19	70	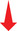	27	5	81.48	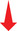
24	16	16	0	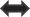	4	4	0	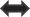
25	56	43	23	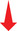	24	18	25	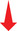
26	104	34	67	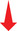	48	3	93.75	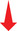
27	57	49	14	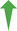	13	15	15.38	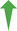
28	82	50	39	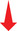	25	15	40	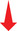
29	37	37	0	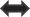	13	13	0	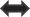
30	112	51	54	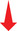	38	14	63.16	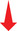
31	35	35	0	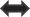	17	17	0	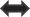
32	38	38	0	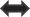	16	16	0	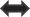
33	31	37	19	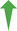	16	17	6.25	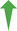
34	30	30	0	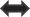	14	14	0	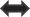
35	46	47	2	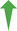	15	16	6.67	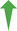
36	45	45	0	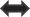	17	17	0	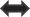
37	68	33	51	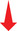	30	10	66.67	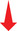
38	33	27	18	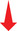	10	7	30	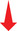
39	50	38	24	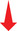	14	9	35.71	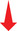
40	22	22	0	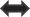	5	5	0	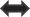
41	33	33	0	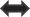	20	20	0	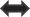
42	89	89	0	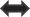	30	30	0	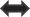
43	42	39	7	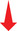	12	10	16.67	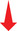
44	65	65	0	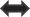	27	27	0	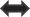
45	63	70	11	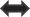	16	16	0	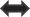
46	41	46	12	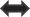	11	11	0	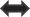
47	34	32	6	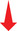	14	13	7.14	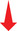
48	171	173	1	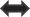	46	46	0	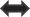
49	111	24	78	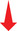	38	8	78.95	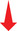
50	44	26	41	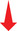	22	11	50	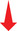
51	34	30	12	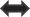	10	10	0	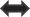
52	90	79	12	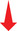	32	26	18.75	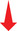
53	43	42	2	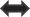	7	7	0	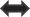
54	73	64	12	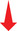	41	38	7.32	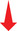
55	41	41	0	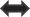	15	15	0	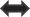
56	56	46	18	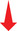	25	17	32	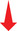
57	43	43	0	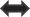	22	22	0	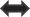
58	51	80	57	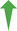	15	26	73.33	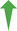
59	534	89	83	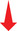	120	17	85.83	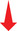
60	33	39	18	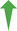	9	11	22.22	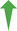
61	78	78	0	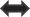	27	27	0	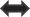
62	83	36	57	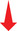	42	8	80.95	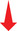
63	35	35	0	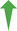	11	13	18.18	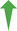
64	32	30	6	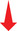	17	4	76.47	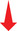
65	74	54	27	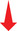	42	28	33.33	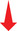
66	54	43	20	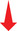	33	29	12.12	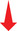
67	54	30	44	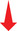	21	10	52.38	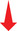
68	47	47	0	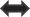	21	21	0	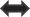
69	52	52	0	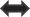	19	19	0	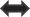
70	55	28	49	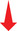	38	10	73.68	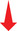
71	52	24	54	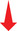	32	10	68.75	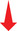
72	56	50	11	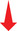	32	26	18.75	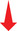
73	63	16	75	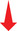	32	5	84.38	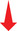
74	30	30	0	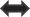	7	7	0	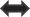
75	68	68	0	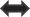	21	21	0	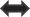
76	48	29	40	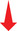	30	9	70	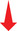
77	44	12	73	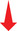	19	6	68.42	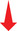
78	83	83	0	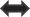	33	33	0	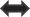
79	73	65	11	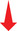	32	26	18.75	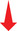
80	66	41	38	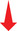	32	16	50	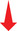
81	931	81	91	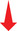	213	16	92.49	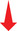
82	59	59	0	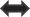	21	21	0	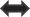
83	77	77	0	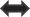	41	41	0	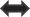
84	17	25	47	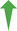	11	13	18.18	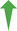
85	63	28	56	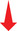	26	9	65.38	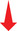
86	42	61	45	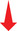	17	16	5.88	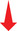
87	57	59	4	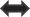	17	17	0	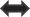
88	76	47	38	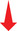	21	12	42.86	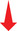
89	67	67	0	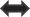	27	27	0	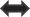
90	38	38	0	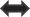	15	15	0	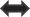
91	34	17	50	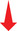	14	4	71.43	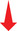
92	35	35	0	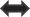	20	20	0	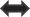
93	27	18	33	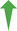	12	4	66.67	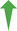
94	84	84	0	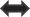	28	28	0	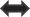
95	42	35	17	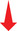	22	20	9.09	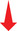

The column ‘OMP No’ refers to the identifier assigned to each analysed outer membrane protein. ‘BAR_E_Count’ and ‘BAS_E_Count’ indicate the number of predicted epitopes for the reference strain (BAR) and the field strain (BAS), respectively. ‘Change Type’ describes the nature of epitope variation (gain, loss or unchanged), while ‘Change (%)’ shows the percentage of this variation relative to the reference. The ‘Directions’ column indicates the direction of change: ‘↑’ for epitope gain, ‘↓’ for epitope loss and ‘↔’ for no change.

As detailed in Table 5, BoLA I epitope prediction analysis revealed peptide count variations in *P39*, *Omp16*, *Omp19*, *Omp25*, *Omp31*, *SodC* and *Glycine betaine/L‐proline* proteins. Peptide losses of 83.74% in *P39*, 34.38% in *Omp16*, and 43.64% in *SodC* were observed. (Supporting Information S5) Conversely, *Omp25* and *Omp31* exhibited peptide increases of 16.64% and 3.27%, respectively. In terms of binding strength, the number of strong‐binding peptides in the BAR strain was identified as 511 for *P39*, 300 for *Omp16*, and 164 for *Omp19*, whereas in the BAS strain, these values were reduced to 105, 216 and 170, respectively. A decrease in strong‐binding peptides was observed in *P39*, *Omp16* and *SodC* in the BAS strain, while an increase was detected in *Omp25* and *Omp31*. The presence of unique peptides suggests distinct immune targets between the BAS and BAR strains. The BAS strain contained 12 unique peptides for *P39* and 47 for *Omp31*. BoLA II epitope prediction analysis indicated peptide losses of 84.98% in *P39*, 35.71% in *Omp16* and 45.28% in *SodC*. Changes of 13.7%, 3.35% and 6.48% were observed in *Omp25*, *Omp31* and *Glycine betaine/L‐proline*, respectively (Supporting Information S6). No changes were detected in *Omp19* (0%). Regarding binding strength, the BAR strain exhibited 889 strong‐binding peptides for *P39*, 346 for *Omp16*, and 168 for *Omp19*. In the BAS strain, these values were 188, 211 and 168, respectively. A reduction in strong‐binding peptides was observed in *P39* and *Omp16* in the BAS strain, whereas *Omp31* showed an increase (Table 5).

**TABLE 5:** This analysis presents the BoLA I and BoLA II binding predictions for BAR and BAS strains.

BoLA I								
BoLA I count type	*P39*	*Omp16*	*Omp19*	*Omp25*	*Omp31*	*SodC*	*Glycine betaine/L‐proline*
**BAR peptide count**	412	160	169	182	245	165	299
**BAR peptide count total**	41,200	16,000	16,900	18,200	24,500	16,500	29,900
**BAS peptide count**	67	105	169	205	253	93	280
**BAS peptide count total**	6700	10,500	16,900	20,500	25,300	9300	28,000
**Change (%)**	83.74% lost	34.38% lost	0	12.64% gain	3.27% gain	43.64% lost	6.35% lost
**BAR—Strong binding**	511	300	164	340	425	228	484
**BAR—Weak binding**	1356	692	469	665	1036	477	1027
**BAS—Strong binding**	105	216	170	307	470	209	454
**BAS—Weak binding**	242	551	473	739	1088	477	953
**BAR unique**	64	30	21	31	44	21	53
**BAS unique**	12	24	22	32	47	17	48

BoLA II								
BoLA II count type	*P39*	*Omp16*	*Omp19*	*Omp25*	*Omp31*	*SodC*	*Glycine betaine/L‐proline*
**BAR peptide count**	3654	1386	1467	1584	2151	1431	2637
**BAR peptide count total**	3654	1386	1467	1584	2151	1431	2637
**BAS peptide count**	549	891	1467	1791	2223	783	2466
**BAS peptide count total**	549	891	1467	1791	2223	783	2466
**Change (%)**	84.98% Lost	35.71% Lost	0	13.7% Gain	3.35% Gain	45.28% Lost	6.48 % Lost
**BAR—Strong binding**	889	346	168	262	620	267	681
**BAR—Weak binding**	2765	1040	1299	1322	1531	1164	1956
**BAS—Strong binding**	188	211	168	373	672	320	651
**BAS—Weak binding**	361	680	1299	1418	1551	463	1815
**BAR unique**	406	154	163	176	239	159	293
**BAS unique**	61	99	163	199	247	87	274

BAR peptide count and BAS peptide count indicate the number of peptides identified in each strain. Change (%) represents the rate of variation, where Lost indicates peptide loss, Gain signifies an increase and Same denotes no change. Strong binding and Weak binding classify peptides based on their binding affinity. Unique values represent peptides exclusively present in the respective strain.

## 4 Discussion

Our study extensively analysed the genomic features of the BAS19 strain, examining its genetic variations and immunogenic potential. Consistent with existing literature, our findings confirm that the *B. abortus* genome is highly conserved while exhibiting significant genetic variations in specific regions (A. Kumar et al. 2022; Islam et al. 2019; Bolotin et al. 2021). This balance between conservation and variation suggests that genomic stability does not preclude the possibility of region‐specific genetic adaptations. The genome length of the BAS19 strain, isolated from Erzurum, was determined to be 3,282,271 bp, showing 99.4% similarity to the reference strain BAR19 (Figure 1). This high similarity reinforces the notion that *B. abortus* genomes remain highly conserved despite regional variations. This finding aligns with previous reports on *B. abortus* biovar 3 isolated from Bangladesh (3,244,234 bp) and *B. abortus* 2308 identified in India (3,285,606 bp) (Islam et al. 2019; A. Kumar et al. 2022). Similarly, Crasta et al. (2008) described the S19 strain as consisting of two circular chromosomes with a total length of 3.283 Mb. This high level of genomic conservation further underscores the evolutionary stability of *B. abortus* genomes. However, comparative genomic analysis indicates that BAS19 harbours unique mutations, insertions and deletions that may contribute to adaptive evolution, host–pathogen interactions, and immune evasion strategies. In addition, comparative genomic analysis revealed that BAS19 contains 1191 more ORFs than BAR19, which may contribute to its enhanced functional capacity and adaptability (Table 1).

The BAS19 strain exhibits significant genetic variations, including 1153 SNPs, 120 insertions and 2501 deletions, primarily concentrated in virulence factors and gene regions influencing immune responses. These genetic alterations may contribute to changes in host–pathogen interactions and bacterial adaptation. Genetic modifications in the S19 strain have been reported, leading to virulence attenuation, with SNPs concentrated in OMPs and metabolic regulatory genes, suggesting their role in virulence and immune response mechanisms. This indicates that while the genome structure remains conserved, phenotypic differences may arise in specific genetic regions (Crasta et al. 2008) (Supporting Information S1). Variant analysis of the BAS19 genome revealed an uneven distribution of SNPs across chromosomes, with 696 on Chromosome 1 and 457 on Chromosome 2 (Figure 3). This asymmetric distribution may indicate localized selective pressures shaping genomic adaptation. The irregular distribution of SNPs and INDELs across BAS19 chromosomes highlights the strain's genetic adaptation to local environmental pressures. These variations may influence regulatory mechanisms associated with host adaptation, a phenomenon previously observed in other *Brucella* species (Mao et al. 2014). Also, these findings may underscore the role of SNPs in genomic differentiation between BAS19 and BAR19, providing insights into local epidemiological dynamics. Particularly, the majority of insertions and deletions were found in genes related to metabolic processes, suggesting potential metabolic flexibility that might enhance BAS19's survival in diverse environmental conditions (Table 3). Ultimately, this study reinforces the significance of SNP analyses in elucidating genomic variations for developing effective protective strategies (Janke et al. 2023; Špičić et al. 2021)

The comparative analysis of the BAS19 genome, evaluated alongside the BAR19 reference genome, *B. abortus* (8 genomes) and *B. melitensis* (18 genomes), demonstrates a notable genetic advantage in terms of subsystem profiles and overall genetic capacity. The BAS19 strain exhibits a subsystem profile encompassing 2485 genes, surpassing the BAR19 reference genome, which contains 2150 genes. This increased genetic repertoire suggests an expanded functional capacity that may enhance environmental adaptability and survival strategies. In comparison, *B. abortus* (8 genomes) harbours only 37 genes, while *B. melitensis* (18 genomes) contains 42 genes within similar subsystems (Elrashedy et al. 2024) (Figure 2). These differences suggest that BAS19 possesses enhanced genetic capacity, contributing to greater biological functionality and environmental adaptability. In particular, genes related to cellular stress response, membrane transport and energy metabolism appear to be enriched in BAS19, supporting its ability to survive in host environments (Monreal et al. 2003). In the stress response, defence, and virulence categories, BAS19 encodes 169 genes, significantly exceeding the 128 genes identified in BAR19 and far surpassing the reported values for *B. abortus* (5 genes) and *B. melitensis* (5 genes) (Elrashedy et al. 2024). (Figure 5). This variation underscores BAS19's optimized regulatory mechanisms for immune response modulation and its enhanced ability to adapt to environmental stress factors, which could impact its pathogenic potential.

In the BAS19 genome annotation, 772 hypothetical and 3717 functional proteins were identified (Figure 2), among which 169 were categorized as virulence factors. In contrast, the BAR19 genome contained 604 hypothetical proteins, 2703 functional proteins, and 128 virulence factors. In addition, 53 tRNA and 9 rRNA genes were identified in BAS19, displaying minor differences compared to BAR19 (55 tRNA, 9 rRNA). Despite these minor variations, the functional implications of these differences warrant further investigation, particularly concerning their role in stress adaptation and host interaction. Bolotin et al. (2021) reported substantial genetic variations in *B. abortus* 68, particularly in genes associated with virulence and metabolic pathways. The BAS19 genome was found to encode a higher number of proteins in both PLFams and PGFams (4279 and 4313, respectively), suggesting greater evolutionary diversity. Furthermore, BAS19 demonstrated increased involvement in stress response, defence and virulence processes (Figure 4). Sternon et al. (2018) highlighted that genomic differences contribute to host adaptation mechanisms, further supporting the observed variations in BAS19. Our study confirms that BAS19 harbours a greater number of virulence factors compared to BAR19 (BAR19: 128, BAS19: 169) (Figure 5). The expanded virulence factor profile of BAS19 highlights its enhanced ability to regulate infection processes and adapt to the host immune system. Among these factors, 30 genes exhibit genetic variations such as insertions and deletions. Notably, *groEL* and *DnaK* play crucial roles in regulating cellular stress responses, supporting bacterial survival under acidic and oxidative conditions (Salmon‐Divon et al. 2018). Omp25 suppresses pro‐inflammatory cytokine production, facilitating chronic infection processes (Degos et al. 2020). *Omp31* promotes autophagy, neutralizing host defence mechanisms (Y. Wang et al. 2021). Asp24 activates the immune system (J. Zhang et al. 2019), while *ExsA* participates in ATP‐binding cassette transport systems, contributing to virulence regulation (Rosinha et al. 2002; Becker et al. 1995; Shrestha et al. 2015). In addition, genes involved in metabolic processes, such as *pgm*, *pgk* and *purE*, are critical for the survival and infection mechanisms of *Brucella* spp. (Y. Zhang, Li, et al. 2016; Trant et al. 2010; Ugalde et al. 2000). These findings emphasize the intricate interplay between virulence factors and metabolic pathways in shaping BAS19's adaptive and pathogenic potential. Moreover, our findings indicate that BAS19 harbours additional transport‐related genes involved in metal ion acquisition, which may facilitate intracellular survival within host cells (Table 6).

**TABLE 6:** Pr3otein and locus indicate the name of the examined protein and its corresponding locus information.

Protein and locus	Signal peptide	Functions	Immunogenic properties	References
***P39*** **(BAbS19_II05150)**	Signal peptide present, cleavage site AA 27 (similarity to SP39_BRUSU)	A solvent‐binding component of bacterial transport systems.	It stimulates the immune system due to its immunogenic properties.	V. Kumar et al. (2018)
***Omp16* (BAbS19_I15960)**	Signal peptide present, cleavage site AA 22 (similarity to BLAB_PROVU)	Lipoprotein found in the cell membrane; activates the immune system.	It triggers the Th1 type immune response and is effective in dendritic cell activation.	Johnson et al. (2019), L. Zhang et al. (2017)
***Omp19* (BAbS19_I18100)**	Signal peptide present, cleavage site AA 20 (similarity to OMP19_BRUSU)	Part of the outer membrane protein transport system.	It increases the immune response by activating CD4+ and CD8+ T cells.	Lee et al. (2021), )
***Omp25*** **(A0A481V8S2)**	Signal peptide present, cleavage site AA 23 (similarity to OM25_BRUSU)	Provides cell membrane stability and plays a role in the bacterial infection mechanism.	It promotes the Th1 type immune response and IgG2a antibody production.	Patel et al. (2017), Chan et al. (2019)
***Omp31* (BAbS19_I15320)**	Signal peptide present, cleavage site AA 27 (similarity to OM31_BRUSU)	Protein that provides stability in the cell membrane; interacts with the immune system.	It triggers the Th1 type immune response and provides immunity after infection.	H. Zhang, Gupta, et al. (2016), Gupta et al. (2020)
***SodC* (BAbS19_II05060)**	Signal peptide present, cleavage site AA 19 (similarity to SODC_BRUAB)	Provides protection against oxidative stress by converting superoxide radicals into harmless compounds.	It is recognized by the host immune system as a virulence factor.	Lin et al. (2018), Zhao et al. (2019)
***Glycine betaine/L‐proline* (BAbS19_II04730)**	Signal peptide present, cleavage site AA 23 (similarity to OMPA_SALTY)	Regulates the transport of glycine betaine and proline from outside the cell to cope with osmotic stress.	It plays a role in metabolic adaptations while carrying osmoprotectants.	J. Zhang et al. (2020), Y. Wang et al. (2021)

Signal peptide specifies whether the protein possesses a signal peptide and identifies its cleavage site. Functions describe the cellular and biochemical roles of the protein. Immunogenic properties explain the protein's impact on the immune system and its immunogenic characteristics. References provide literature citations related to the studied protein. Proteins with a signal peptide are marked in bold.

Beta‐barrel proteins are integral to the outer membrane of *Brucella* spp., playing a pivotal role in infectivity, immune evasion and membrane stability. In the BAS19 strain, their prevalence declined from 34.7% to 24.2%, with the loss of nine proteins, suggesting a strategic shift in infection dynamics. This reduction likely represents an evolutionary trade‐off between structural integrity and immune evasion. Notably, beta‐barrel proteins are associated with a diminished inflammatory response (Degos et al. 2020) and enhanced membrane stability (Godessart et al. 2020). Their depletion in BAS19 may, therefore, facilitate immune escape (Valguarnera et al. 2018). In particular, the reduction or loss of beta‐barrel structures in BAbS19_I00090, BAbS19_I09030, BAbS19_II02220 and BAbS19_II10320 could significantly modulate immune evasion mechanisms, thereby reshaping host–pathogen interactions and contributing to the strain's adaptive evolution (Singh et al. 2020; Audic et al. 2009; Cabello et al. 2024; Pei et al. 2023) (Table 2). Furthermore, the loss of specific beta‐barrel proteins correlates with altered membrane permeability, which may have implications for antibiotic resistance (Table 5).

The observed protein structural changes in the BAS19 strain could have multiple effects: i) They may reduce virulence while optimizing or improving immune responses (Monreal et al. 2003; Caro‐Hernández et al. 2007; Crasta et al. 2008; S. Wang et al. 2020). ii) They might weaken immune responses and negatively impact bacterial adaptation to environmental stresses (Pasquevich et al. 2008; Solanki et al. 2020). iii) They could enhance protective immunity against the pathogen (Edmonds et al. 2002; Grilló et al. 2006; Yang et al. 2010; Paul et al. 2018). Epitope mapping in our study provides initial insights into these changes. According to BepiPred and ABCpred analyses, 61.05% of the total proteins exhibited epitope variations, with 49.47% showing epitope loss and 11.58% acquiring new epitopes Table 4, literature reports indicate that epitope losses in immunogenic proteins such as *Omp25*, *Omp31*, *Omp2b* and *BCSP31* could enhance immune evasion strategies, potentially increasing chronic infection potential (Yang et al. 2020; Shi et al. 2023). Conversely, epitope acquisitions have been reported to enhance immunogenic potential (Ren et al. 2021). The significant epitope loss in *P39* suggests that it may induce an immune evasion mechanism against BoLA binding, reducing its effectiveness as an immunological target (Lamontagne et al. 2010). The loss in *Omp16* is predicted to impair antigen presentation (Velásquez et al. 2017). Similarly, the losses observed in *SodC* and *Glycine Betaine/L‐proline* proteins are considered critical for immune evasion (Milillo et al. 2019). The decrease in *DnaK* could reduce bacterial interactions with the immune system (S. Wang et al. 2020). A notable 82.14% reduction in *P39* suggests significant immune escape potential, whereas functional gains were observed in genes with increased length, such as *Omp25* (Escalona et al. 2017), *cp24*, *asp24* and *Glyoxalase* (Sheehan et al. 2020). However, these adaptations may make it more difficult for the host immune system to target the strain effectively. The absence of length variations in *Omp19* and *VjbR* indicates that these proteins have retained their core virulence functions (Zai et al. 2017) (Table 3).

Signal peptide analysis of *B. abortus* revealed that seven out of the 30 examined proteins harbour signal peptides, suggesting their targeting to organelles such as the endoplasmic reticulum (ER). These proteins potentially modulate immune responses, underscoring their significance in host–pathogen interactions (Pasquevich et al. 2019). Notably, OMPs including *P39*, *Omp16*, *Omp19*, *Omp25*, *Omp31*, *SodC* and *Glycine Betaine/L‐proline* exhibit immunogenic properties, which may elicit robust immune responses (Farahi et al. 2012) (Table 6). These observations indicate that these proteins could serve as viable immunological targets and contribute to virulence mechanisms (Jade et al. 2023). The presence of signal peptides likely enhances the initiation of immune responses, thereby playing a pivotal role in the pathogenesis and immune evasion strategies of *B. abortus* (Sung et al. 2014). In addition, *B. abortus* modulates host immune responses by inducing ER stress, a strategic mechanism facilitating bacterial survival (Guimarães et al. 2023; Byndloss et al. 2019; Guimarães et al. 2019; Tsai et al. 2022). Collectively, our findings elucidate the dynamic genomic architecture of BAS19, providing insights into its sophisticated immune evasion tactics, metabolic adaptability and implications for *Brucella* pathogenesis. These findings hold promise for advancing vaccine design and targeted therapeutic interventions.

## 5 Conclusion

This study provides a comprehensive analysis of the genomic and immunogenic characteristics of the BAS19 strain, comparing it to the BAR19 reference genome and other *Brucella* strains, thereby identifying key genetic and functional differences. The findings indicate that while the genomic structure of BAS19 remains largely conserved, genetic variations such as SNPs, insertions and deletions are predominantly concentrated in virulence factors and immune response‐associated gene regions. The broader repertoire of virulence factors in BAS19 compared to BAR19 suggests that the strain has developed genetic adaptations that may enhance its infection capacity. Notably, the reduction in beta‐barrel protein levels and the complete loss of nine proteins indicate potential reshaping of *Brucella*’s immune evasion mechanisms. Epitope analysis revealed that epitope losses (49.47%) and gains (11.58%) in BAS19 significantly alter immune recognition processes. In addition, beta‐barrel protein reductions and BoLA epitope variations highlight potential implications for protective immunity. Ultimately, the genomic adaptations of BAS19 offer a valuable model for understanding the evolutionary dynamics of *Brucella* infections. The immunogenic potential of BAS19 provides critical insights for therapeutic targets and vaccine strategies, emphasizing the necessity for long‐term assessments of live attenuated vaccine strains to evaluate their protective efficacy.

## Author Contributions

**Ali Arslan**: writing – review and editing, writing – original draft, visualization, methodology, conceptualization. **Emre Aktas**: methodology, data curation. **Osman Ugur Sezerman**: investigation, writing – review and editing. **Tulin Ozbek**: supervision, writing – review and editing, project administration.

## Funding

This work was supported by the Yildiz Technical University Scientific Research Projects Coordination Unit, Project No: FDK‐2022‐5166.

## Conflicts of Interest

Ali Arslan, Tülin Özbek, Osman Uğur Sezerman and Emre Aktaş declare that they have no conflict of interest in this study.

## Peer Review

The peer review history for this article is available at https://www.webofscience.com/api/gateway/wos/peer‐review/10.1002/vms3.70593.

## Supporting information




**Supplementary File 1**: vms370593‐sup‐0001‐SuppMat1.xlsx

**Supplementary File 2**: vms370593‐sup‐0002‐SuppMat2.docx

**Supplementary File 3**: BAS strain colonies grown on blood agar. The colonies appear as small, round, and smooth white dots distributed across the agar surface.

**Supplementary File 4**: List of OMPs identified in the BAS strain. The table provides the corresponding Protein IDs and Locus tags, indicating the genomic locations of these OMPs within the BAS strain.

**Supplementary File 5**: vms370593‐sup‐0005‐SuppMat5.xlsx

**Supplementary File 6**: vms370593‐sup‐0006‐SuppMat6.xlsx

## Data Availability

The data that support the findings of this study are openly available in the NCBI database under the reference number SRR14268008.
