# Supplementary figures and images for: Genomic and Immunoinformatics Insights Into a Bovine‐Derived Brucella abortus S19 Field Strain: Adaptations Impacting Vaccine Efficacy
Source: Vet Med Sci. 2025 Nov 3;11(6):e70593. doi: 10.1002/vms3.70593 (PMC12581176; doi:10.1002/vms3.70593)

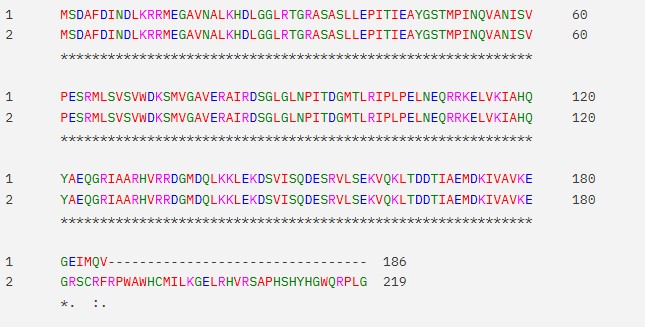


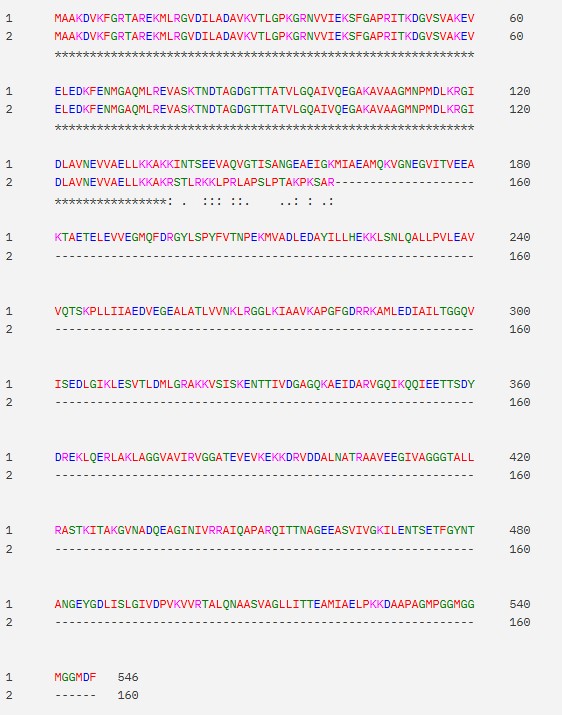


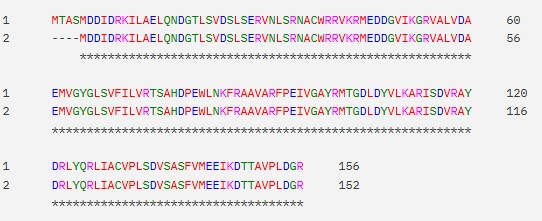


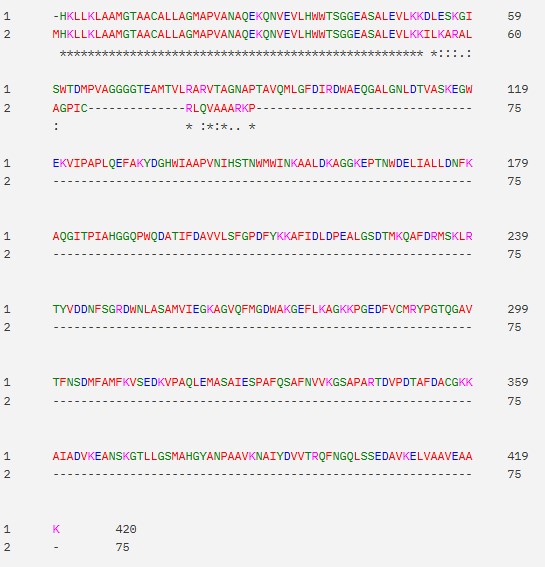


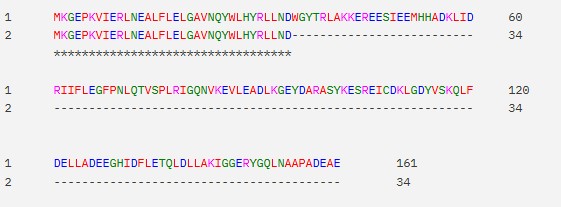


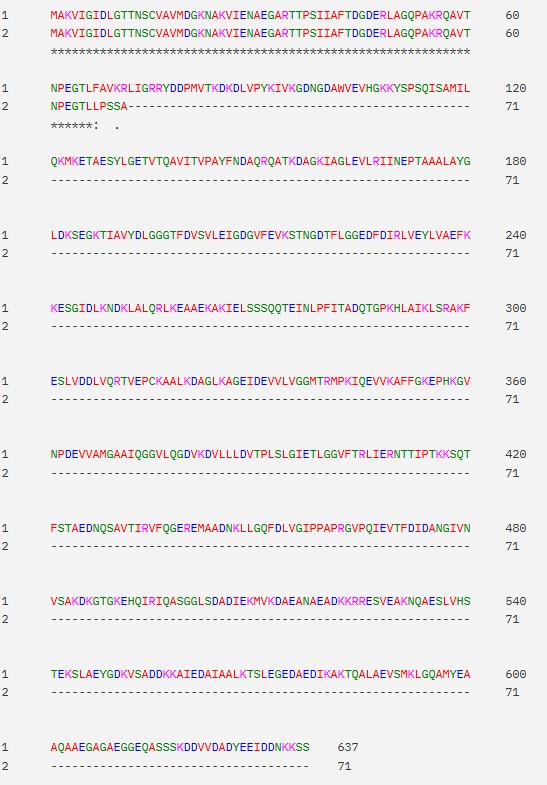


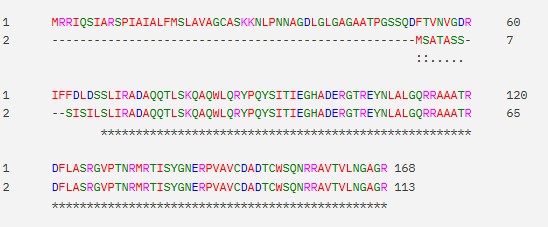


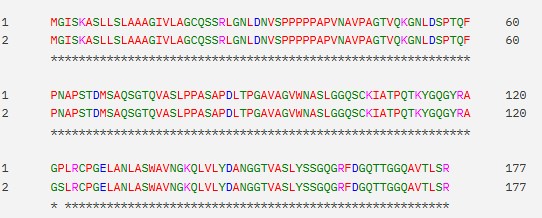


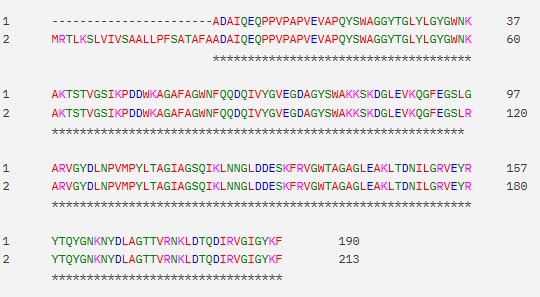


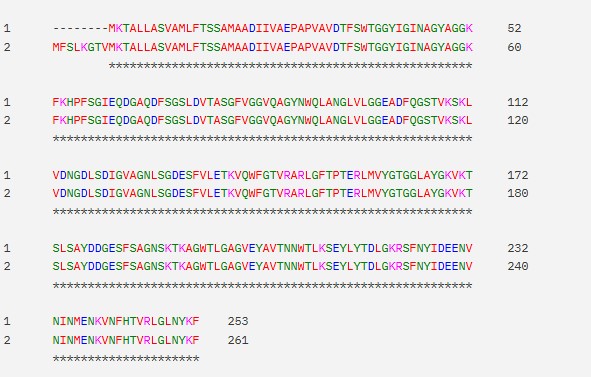


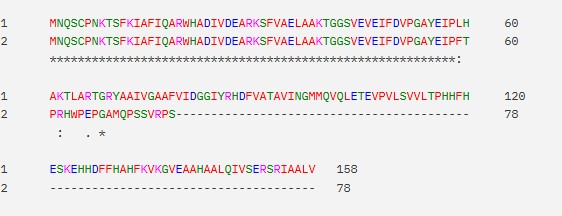


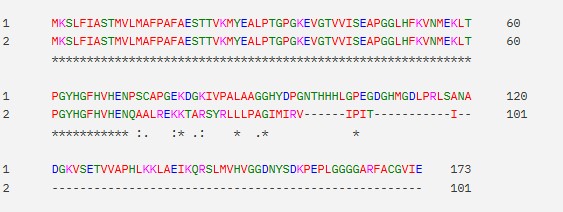


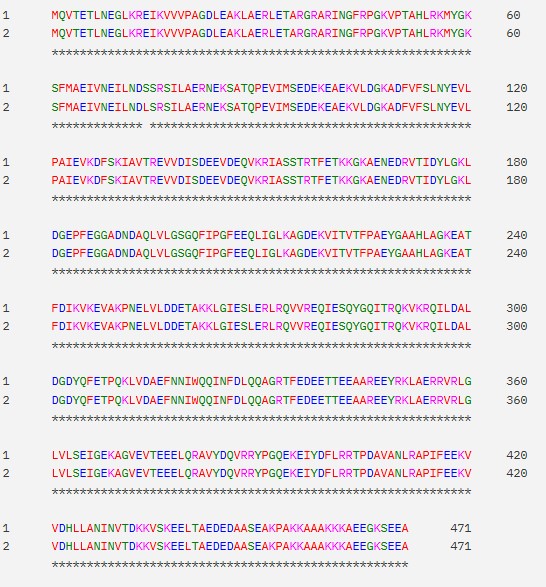


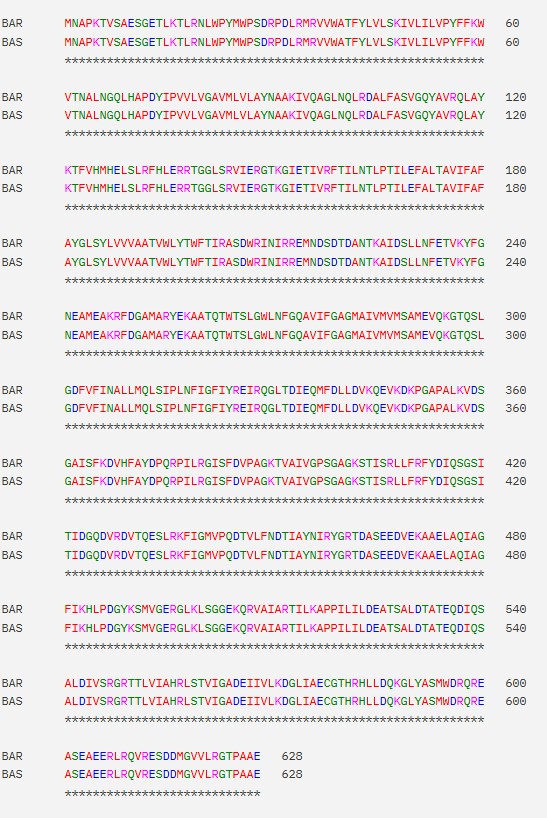


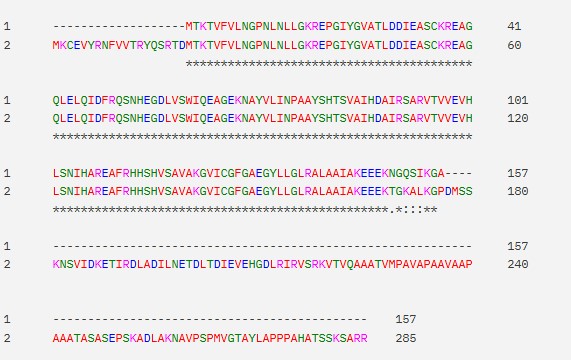


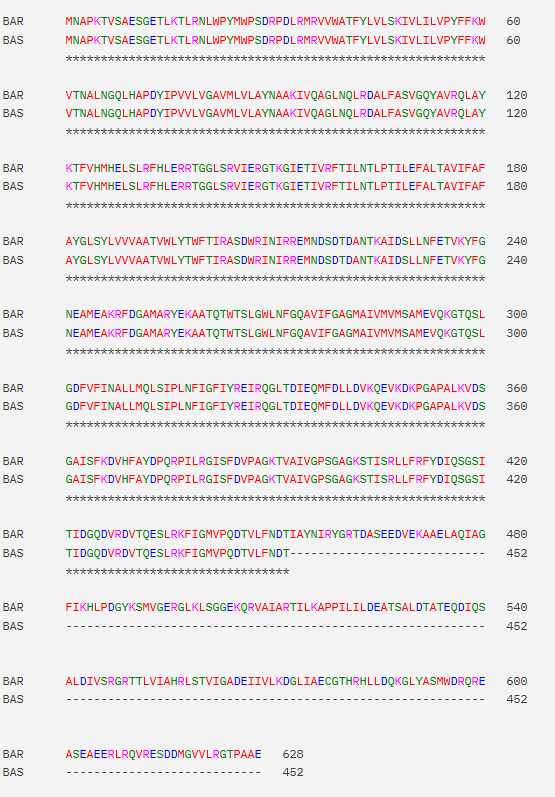


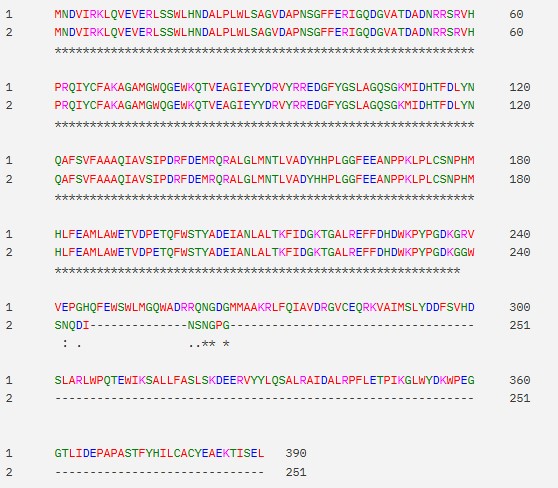


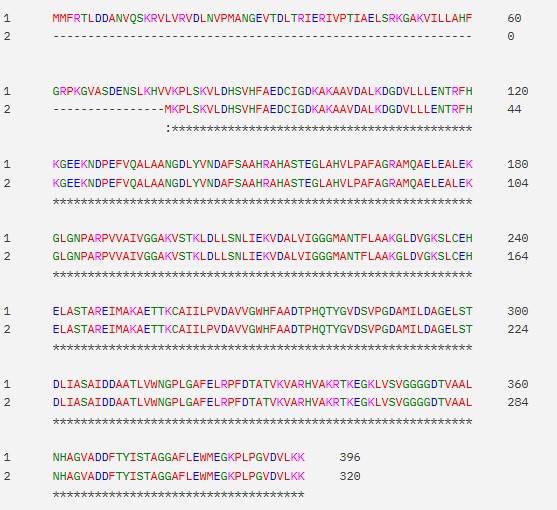


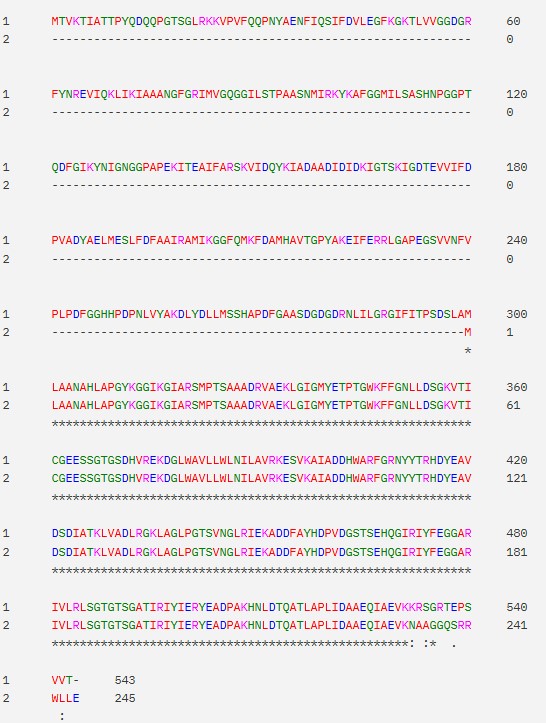


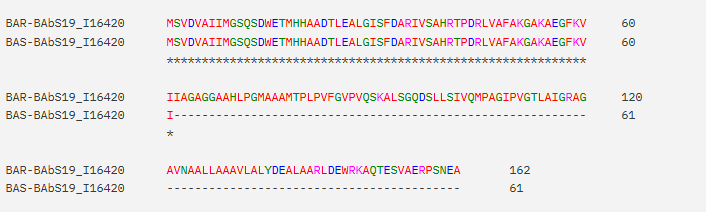


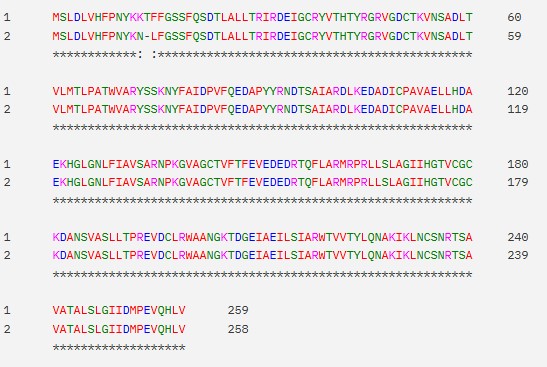


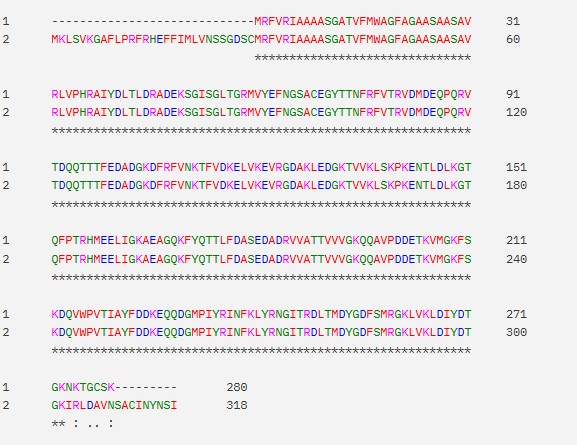


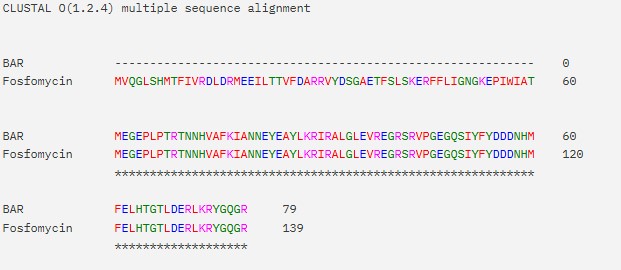


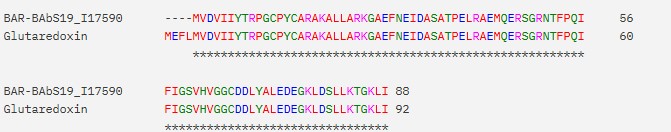


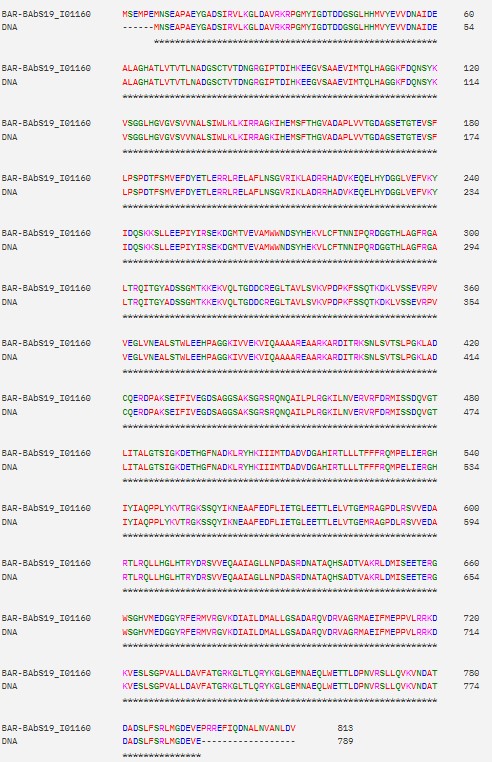


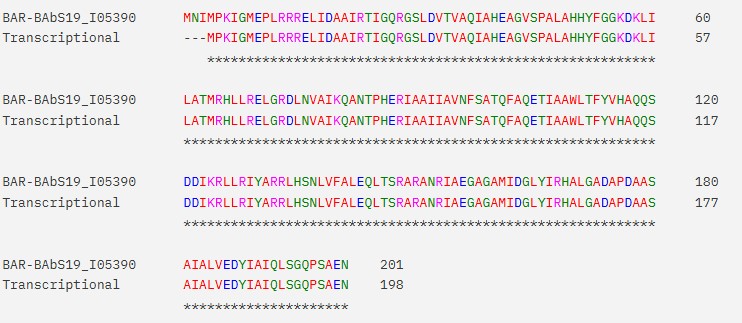


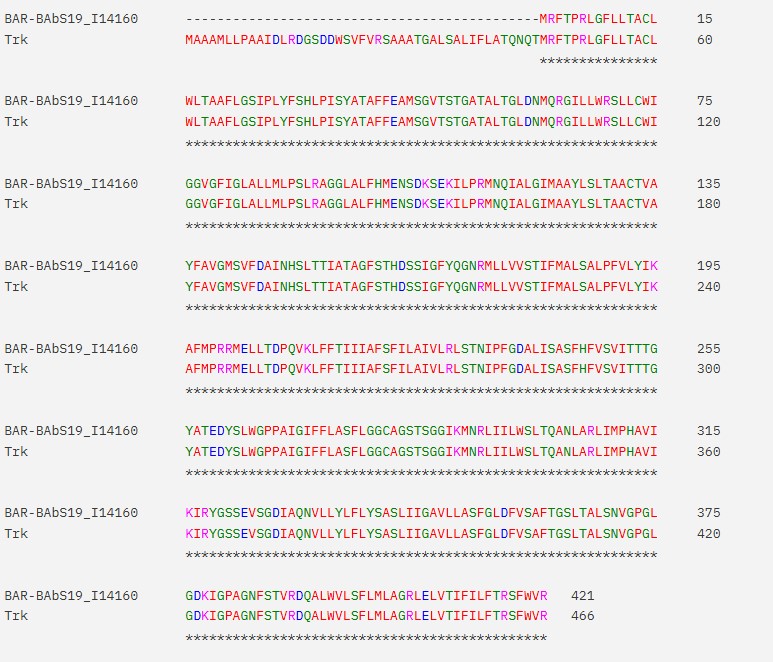


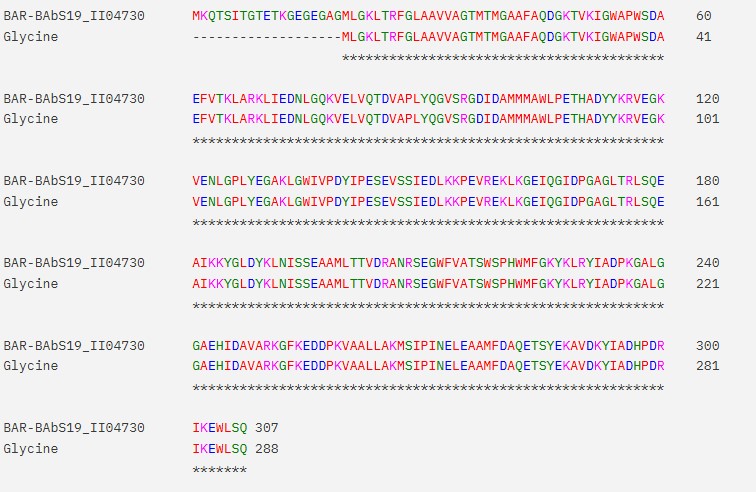


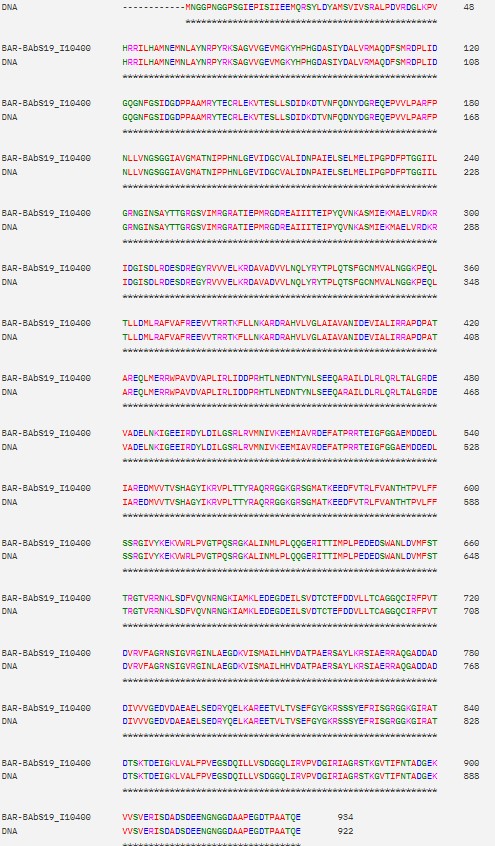


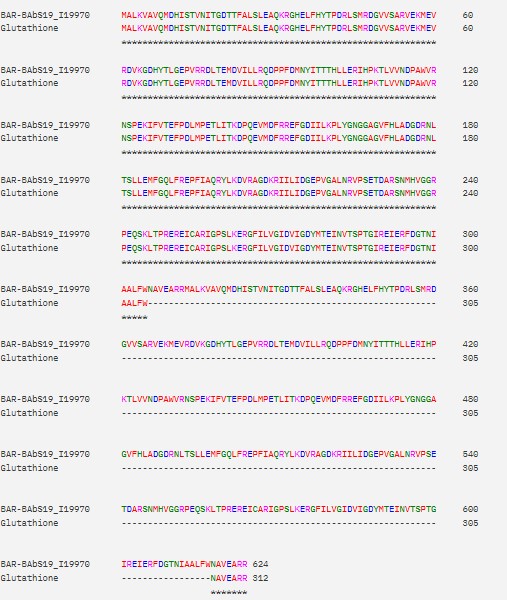


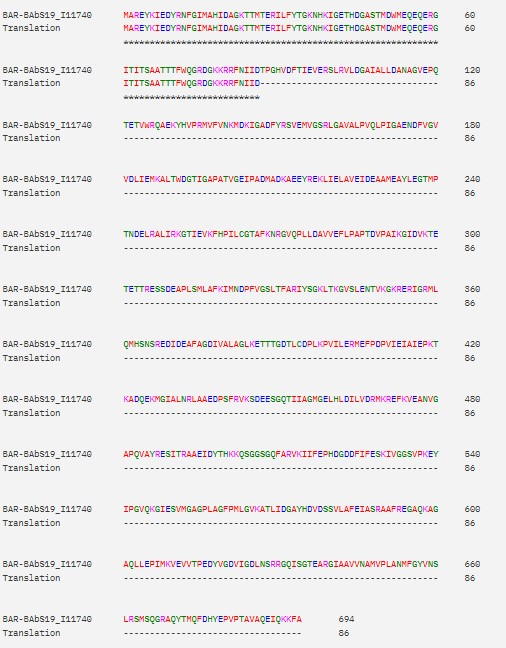

Supplement: Supplementary file 2 — vms370593‐sup‐0002‐SuppMat2.docx [file VMS3-11-e70593-s006.docx]

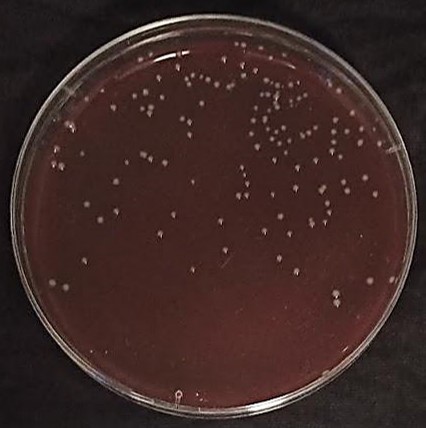

Supplement: Supplementary file 3 — BAS strain colonies grown on blood agar. The colonies appear as small, round, and smooth white dots distributed across the agar surface. [file VMS3-11-e70593-s005.jpg]

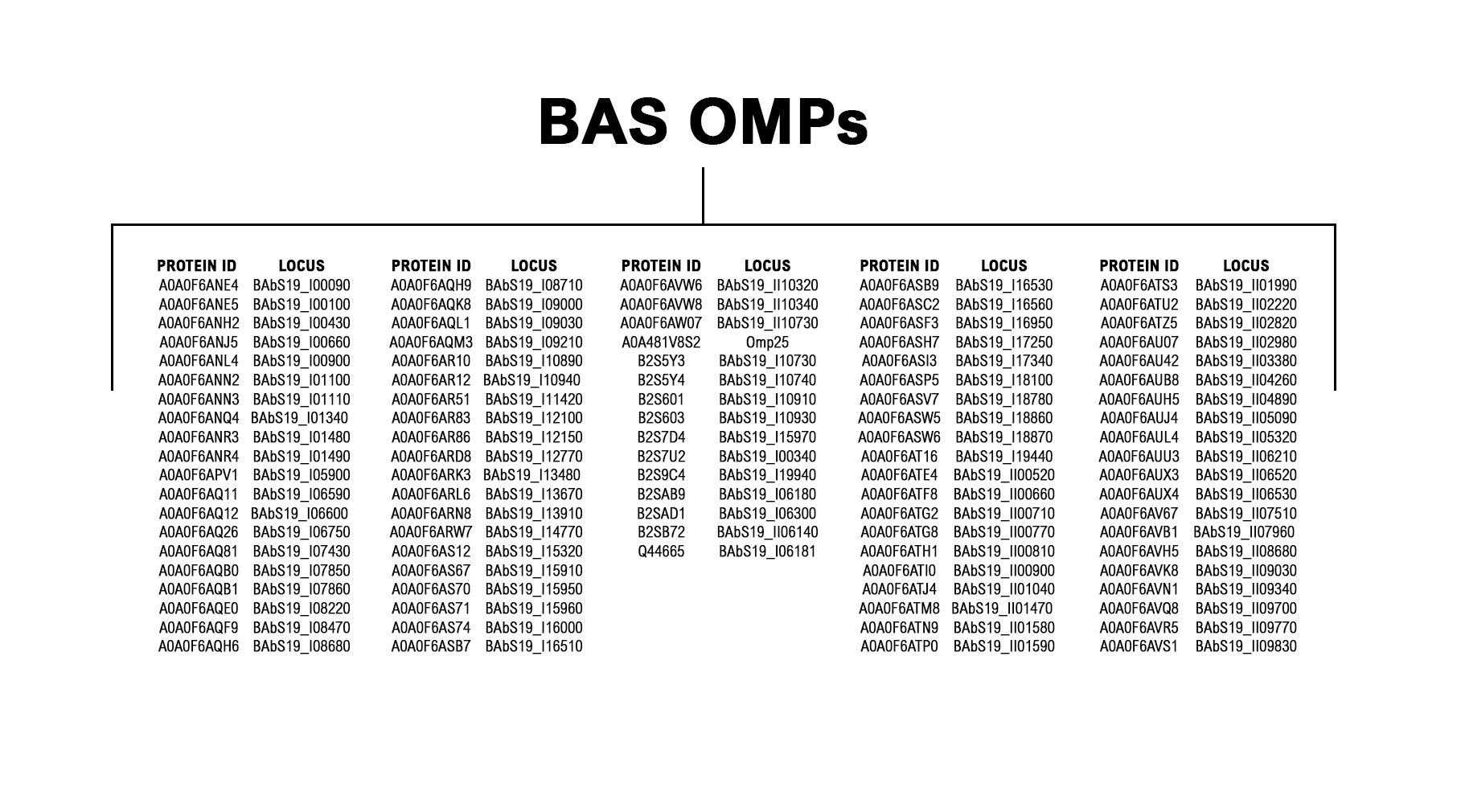

Supplement: Supplementary file 4 — List of OMPs identified in the BAS strain. The table provides the corresponding Protein IDs and Locus tags, indicating the genomic locations of these OMPs within the BAS strain. [file VMS3-11-e70593-s001.jpg]
